# Untargeted metabolomics identifies metabolic dysregulation of sphingolipids associated with aggressive chronic lymphocytic leukaemia and poor survival

**DOI:** 10.1002/ctm2.1442

**Published:** 2023-11-30

**Authors:** Flora Nguyen Van Long, Délya Valcourt‐Gendron, Patrick Caron, Michèle Rouleau, Lyne Villeneuve, David Simonyan, Trang Le, Roxanne Sergerie, Isabelle Laverdière, Katrina Vanura, Chantal Guillemette

**Affiliations:** ^1^ Centre Hospitalier Universitaire de Québec Research Center‐Université Laval (CRCHUQc‐UL) Faculty of Pharmacy and Centre de Recherche sur le Cancer (CRC‐UL) Université Laval Québec Canada; ^2^ Statistical and Clinical Research Platform CRCHUQc‐UL Québec Canada; ^3^ Department of Medicine I Division of Haematology and Haemostaseology Medical University of Vienna Vienna Austria; ^4^ Canada Research Chair in Pharmacogenomics Québec Canada

**Keywords:** chronic lymphocytic leukaemia, drug target, lipidomics, metabolomics, prognostic marker, sphingolipids

## Abstract

**Background:**

Metabolic dependencies of chronic lymphocytic leukaemia (CLL) cells may represent new personalized treatment approaches in patients harbouring unfavourable features.

**Methods:**

Here, we used untargeted metabolomics and lipidomics analyses to isolate metabolomic features associated with aggressive CLL and poor survival outcomes. We initially focused on profiles associated with overexpression of the adverse metabolic marker glycosyltransferase (UGT2B17) associated with poor survival and drug resistance.

**Results:**

Leukaemic B‐cell metabolomes indicated a significant perturbation in lipids, predominantly bio‐active sphingolipids. Expression of numerous enzyme‐encoding genes of sphingolipid biosynthesis pathways was significantly associated with shorter patient survival. Targeted metabolomics further exposed higher circulating levels of glucosylceramides (C16:0 GluCer) in CLL patients relative to healthy donors and an aggressive cancer biology. In multivariate analyses, C16:0 GluCer and sphinganine were independent prognostic markers and were inversely linked to treatment‐free survival. These two sphingolipid species function as antagonistic mediators, with sphinganine being pro‐apoptotic and GluCer being pro‐proliferative, tested in leukemic B‐CLL cell models. Blocking GluCer synthesis using ceramide glucosyltransferase inhibitors induced cell death and reduced the proliferative phenotype, which further sensitized a leukaemic B‐cell model to the anti‐leukaemics fludarabine and ibrutinib in vitro.

**Conclusions:**

Specific sphingolipids may serve as prognostic markers in CLL, and inhibiting enzymatic pathways involved in their biosynthesis has potential as a therapaeutic approach.

## Graphical abstract

- An investigation was carried out to establish a comprehensive metabolomic profiling of leukaemic B cells with a focus on portraying metabolic features associated with aggressive chronic lymphocytic leukaemia (CLL).
- Specific sphingolipid species, C16:0 glucosylceramide and sphinganine, were identified as novel biomarkers of CLL, and were inversely linked to aggressive disease and treatment‐free survival.
- Our work further emphasizes the potential of inhibiting the main enzymatic pathway involved in C16:0 glucosylceramide biosynthesis as potential therapeutic target in CLL.

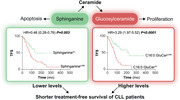

## 1 INTRODUCTION

Chronic lymphocytic leukaemia (CLL) is a highly heterogeneous disease in terms of clinical presentation and course.^1^ Metabolic rewiring is a critical hallmark of tumourigenesis and is essential for the development of cancer and its progression.^2^ This process supports leukaemic cell growth and survival and has been linked to aggressive disease, drug resistance and relapse.^3^, ^4^, ^5^, ^6^, ^7^, ^8^ Metabolomics studies have become a useful tool for discovering novel biomarkers and identifying additional therapeutic targets,^9^ but have not been thoroughly applied to CLL. A few critical metabolic processes appear to be reprogrammed in leukaemic B cells and include glycolysis, lipid metabolism and oxidative phosphorylation, some of which are correlated with aggressive features such as the immunoglobulin heavy‐chain variable (IGHV) mutational status.^3^, ^4^, ^5^, ^6^, ^10^, ^11^, ^12^

Such heterogeneity in the metabolism of CLL cells could be therapeutically exploited. For instance, an abundance of reactive oxygen species and an increased mitochondrial respiration rate are present in CLL cells as compared with normal B lymphocytes, supporting on‐going phase II clinical trials using the mitochondrial respiration inhibitor metformin, alone or in combination with ritonavir, an HIV protease inhibitor that blocks glucose transporters (NCT01750567; NCT02948283).^13^, ^14^ Lipid rewiring also represents a metabolic characteristic of CLL cells, which includes an enhanced dependency on fatty‐acid oxidation.^3^, ^5^, ^15^ Lipases and phospholipases are also overexpressed in CLL cells, such as the lipoprotein lipase, responsible for the hydrolysis of triglycerides into free‐fatty acids. Lipoprotein lipases represent as adverse markers overexpressed particularly in IGHV‐unmutated leukaemic cells, which are associated with an unfavourable prognosis.^16^ Yet, there are only a few reports in which researchers have sought to identify metabolic rewiring associated with aggressive CLL using untargeted metabolomics approaches.^5^, ^17^, ^18^, ^19^, ^20^

For this study, it was our goal to establish a comprehensive profiling of leukaemic B‐cell metabolomes using untargeted metabolomics and lipidomics approaches in leukaemic B‐cell models. We focused on portraying the metabolic features of cancer cells associated with overexpression of the adverse metabolic marker glycosyltransferase (UGT2B17). Upregulated *UGT2B17* expression represents an independent adverse prognostic marker associated with shorter treatment‐free survival (TFS) and shorter overall survival (OS) of CLL patients, as well as poor drug response.^21^, ^22^, ^23^, ^24^ UGT2B17 belongs to a superfamily that comprises 22 enzymes responsible for the conjugation of a vast array of molecules ranging from endogenous metabolites, such as steroids to anti‐cancer agents, linking them to glucuronic acid, which leads to their inactivation.^25^ UGT2B17 affects bio‐active lipids, such as prostaglandin E2, through glucuronidation that impairs their anti‐oncogenic properties with subsequent effects on B‐cell proliferation, thereby contributing to disease progression in CLL patients overexpressing *UGT2B17*.^23^ In multivariate analyses, high leukaemic expression of *UGT2B17* further improves the prognostication of individuals with CLL with either mutated (M‐CLL) or unmutated (UM‐CLL) IGHV,^21^, ^22^, ^23^ suggesting a potential impact on B‐cell metabolism in both CLL subgroups. Furthermore, these associations are independent of other markers including the metabolic marker lipoprotein lipase,^21^ strengthening the likelihood of discovering novel metabolic features of aggressive CLL. Our work identifies sphingolipids as novel biomarkers of CLL and aggressive disease and emphasizes the potential of inhibiting the main enzymatic pathway involved in their biosynthesis as a potential therapeutic target in CLL.

## 2 MATERIALS AND METHODS

### 2.1 Chemicals

All chemicals were high‐performance liquid chromatography grade and are described in Supporting Information.

### 2.2 CLL patient cells, cohorts and healthy donors

A cohort of 107 CLL cases and cryopreserved peripheral blood mononuclear cells from three CLL patients diagnosed between 1987 and 2011 at Vienna General Hospital for whom information on UGT2B17 expression and plasma samples were available,^26^ were studied. CLL diagnosis, staging and requirement for therapy were based on the NCI‐WG2008 guidelines, as described.^26^ CLL patients were early stage and untreated at the time of blood collection, as previously reported.^26^ Patients’ characteristics are provided in Supporting Information Table S1. Plasma and serum samples from 70 healthy donors consisting of 44 women and 26 men were collected at the Centre Hospitalier Universitaire de Québec Research Center—Université Laval (CRCHUQc‐UL) or the Medical University of Vienna, or were purchased from BioIVT (Westbury). B‐CLL cells from 19 CLL cases from the Quebec Leukemia Cell Biobank (BCLQ; https://bclq.org/) were analysed for metabolite content. We also studied four public datasets to assess the relationship between OS and expression profiles of candidate genes including the International Cancer Genome Consortium (ICGC; n = 294 CLL cases), the Broad Institute dataset (n = 156; https://www.cbioportal.org/) and two others available via the online Gene Expression Omnibus (http://www.ncbi.nlm.nih.gov/geo/) under accession number GSE22762 (n = 151) and GSE13159 (n = 448). This research was performed in accordance with the Helsinki Declaration, and the study was evaluated and approved by local Ethical Research Committees of the Medical University of Vienna (Ethics vote 2176/2017) and CHUQc—Université Laval (#2015‐1205).

### 2.3 Metabolomics and lipidomics assays

Metabolomics analyses were performed by Metabolon Inc. on five biological replicates for each cell model (80 × 106 cells), as described previously.^27^ For these experiments, we used MEC1 and JVM2 cells that were previously engineered to overexpress UGT2B17 (UGT2B17^OE^) and their respective control cell line.^23^ Fold changes for metabolites and lipids were calculated based on the mean values, and ANOVA tests were performed to identify those that differed significantly between experimental groups. Metabolon in‐source tools were used for metabolite identification, and to perform pathway enrichment analysis and enrichment score, as described in **Supporting Information**. Specific sphingolipid metabolites were measured by liquid chromatography coupled to tandem mass spectrometry using specimens from CLL patients or healthy donors (25 μL) and cell models (1 × 106 cells), as described in **Supporting Information**.

### 2.4 Cell models and culture

Three leukaemic B‐cell models (JVM2, MEC1 and HG3) were purchased from DSMZ (Braunschweig). Cell lines (MEC1 and JVM2) engineered to overexpress UGT2B17 (UGT2B17^OE^) were described previously.^23^ The HG3‐UGT2B17 knockout line (HG3‐UGT2B17^KO^) was generated by the CRISPR/Cas9 gene mutagenesis as described in **Supporting Information**.

For cell treatment with sphingolipids, C16:0 glucosylceramide (C16:0 GluCer) and sphinganine were dissolved in vehicle consisting of CHCL_3_/MeOH (1:1) and MeOH, respectively. Cells were seeded at a density of 5 × 105 cells/mL and were treated for 48 h at 37°C with C16:0 GluCer or sphinganine (10 μM each). UDP‐glucose ceramide glucosyltransferase (UGCG) inhibitors (UGCGi) eliglustat and ibiglustat and anti‐leukaemics ibrutinib and fludarabine were prepared in DMSO (Sigma–Aldrich). Cells were treated for 96 h with UGCGi at various concentrations, alone or in combination with ibrutinib (1 μM) or fludarabine (10 μM). Additional cell‐based assays and expression analyses are described in **Supporting Information**.

### 2.5 Statistical analysis

Characteristics of the CLL patients were presented as frequencies for categorical variables and as medians with 95% confidence intervals (95% CI) for continuous variables. Clinical and molecular features were compared by sex and expression status, using Pearson's chi‐square test. Metabolite levels were presented as means with standard errors. Sphingolipid concentrations in relation to clinical characteristics were compared using the non‐parametric Mann–Whitney test. The non‐parametric Spearman test was used for correlation analysis between sphingolipid levels measured in CLL patients. Kaplan–Meier survival curves were used to estimate OS or TFS depending on the cohorts, and the log‐rank test (LRT) was used to compare survival curves. Univariate and multivariate analyses were performed using Cox's proportional hazard model, with the adjusted model using IGHV mutational status, 11q deletion, CD38 expression and Binet stage as co‐variables, as described.^26^ The Schoenfeld residuals analyses and the Supremum tests were used to verify that each co‐variable independently satisfied the assumptions of the Cox model. For public datasets, comparisons of median gene expression involved in sphingolipid metabolism according to *UGT2B17* expression were performed using the non‐parametric Mann–Whitney test. The median expression of *UGT2B17* was used as a cut‐off to stratify CLL patients expressing high and low levels of *UGT2B17*. Comparison of mean values between two groups was carried out using a two‐tailed Student's *t*‐test; comparisons for more than two groups were made with a one‐way ANOVA. All *p*‐values were two‐tailed. A value of *p* < .05 was considered statistically significant, and .05 ≤ *p* < .1 indicated a trend. Statistical analyses were performed using SAS version 9.4. (SAS) and GraphPad Prism version 9.5 (GraphPad Software).

## 3 RESULTS

### 3.1 Leukaemic B cells exhibit broad metabolic alterations with changes in sphingolipid metabolism

We first examined metabolic perturbations caused by overexpression of *UGT2B17* associated with aggressive disease and shorter survival^21^, ^22^, ^23^, ^24^ in two leukaemic cell lines using untargeted metabolomics approaches. Compared with control cells expressing low levels, broad changes in cellular levels of metabolites were observed for cells overexpressing UGT2B17 (Table 1). Variations in metabolite concentrations affected 57% of all 496 measured metabolites in JVM2 cells, consistent with the fact that these cells express significantly higher levels of UGT2B17 as compared with MEC1 cells,^23^ in which 29% of measured metabolites were altered. Pathway enrichment analysis identified superpathways similarly altered in both cell lines including lipids, nucleotides, carbohydrates, and co‐factors and vitamins (Supporting Information Table S2).

**TABLE 1:** Metabolomic alterations associated with the metabolic prognostic marker UGT2B17 in leukaemic B‐cell lines based on untargeted metabolomics and lipidomics.

Altered metabolites	MEC1^OE vs CTRL^ n (%)	JVM2^OE vs CTRL^ n (%)
UNTARGETED METABOLOMICS		
Metabolites identified	496	496
Number of altered metabolites		
Total metabolites	142 (28.6%)	280 (56.5%)
Increased	71 (14.3%)	97 (19.6%)
Decreased	71 (14.3%)	183 (36.9%)
Number of significantly altered metabolites		
Total metabolites	98 (19.8%)	234 (47.2%)
Increased	51 (1.3%)	80 (16.1%)
Decreased	47 (9.5%)	154 (31.1%)
Number of altered metabolites (trend)		
Total metabolites	44 (8.9%)	46 (9.3%)
Increased	20 (4.0%)	17 (3.4%)
Decreased	24 (4.8%)	29 (5.8%)
LIPIDOMICS		
Metabolites identified	1053	1053
Number of altered metabolites		
Total metabolites	131 (12.4%)	349 (33.1%)
Increased	34 (3.2%)	229 (21.7%)
Decreased	97 (9.2%)	120 (11.4%)
Number of significantly altered metabolites		
Total metabolites	88 (8.4%)	223 (21.2%)
Increased	18 (1.7%)	130 (12.3%)
Decreased	70 (6.6%)	93 (8.8%)
Number of altered metabolites (trend)		
Total metabolites	43 (4.1%)	126 (12.0%)
Increased	16 (1.5%)	99 (9.4%)
Decreased	27 (2.6%)	27 (2.6%)

Cell lines overexpressing (OE) the UGT2B17 protein were compared with their respective control cells (CTRL).

**FIGURE 1:**
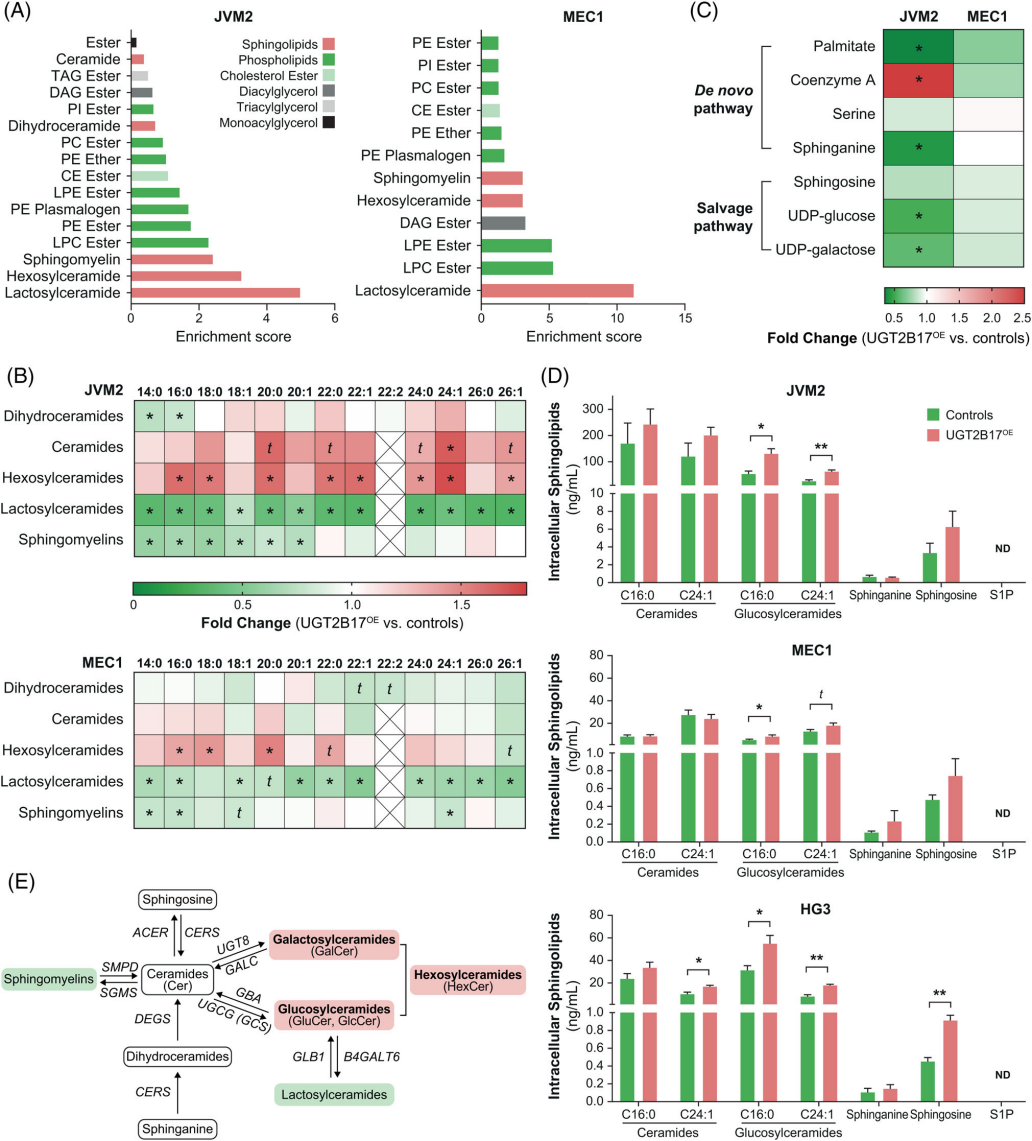
Untargeted and targeted metabolomics indicate that leukemic B‐cell lines expressing high levels of the adverse marker UGT2B17 have broad metabolic alterations and higher production of specific ceramide species. (A) Lipid metabolism pathways that were most substantially altered in JVM2 and MEC1 cells overexpressing UGT2B17 (UGT2B17^OE^) compared to control cells (CTRL). (B, C) Sphingolipid changes were noted in JVM2 and MEC1 cells using lipidomics (B) and untargeted metabolomics (C). *t* (trend): .05 ≤ *p* < .10; *p < .05; **p < .01. Detailed quantitative data from five independent replicates are provided in Supporting Information Table S3. (D) Targeted metabolomics was used to provide quantitative sphingolipid levels in JVM2, MEC1 and HG3 cell lines expressing high levels of UGT2B17 compared to those expressing low or undetectable levels. Data are expressed as the mean ± standard error and are derived from at least three independent experiments. (E) Schematic representation of the main findings providing evidence of an accumulation (highlighted in red) of hexosylceramides and lower levels (highlighted in green) of sphingomyelins and lactosylceramides. Biosynthetic pathways responsible for the interconversion of sphingolipids and the genes coding for the enzymes (in italics) are also indicated. TAG, triacylglycerol; DAG, diacylglycerol; CE, cholesterol ester; PI, phosphatidylinositol; PC, phosphatidylcholine; PE, phosphatidylethanolamine; LPE, lysophosphatidylethanolamine; LPC, lysophosphatidylcholine; N/D, not detected; *ACER*, alkaline ceramidase; *SGMS*, sphingomyelin synthase; *SMPD*, sphingomyelin phosphodiesterase; *GBA*, glucosylceramidase beta; *GLB1*, galactosidase beta 1; *B4GALT6*, beta‐1,4‐galactosyltransferase 6.

To investigate these changes further, we performed an untargeted lipidomics analysis (Table 1). Among the top five most altered lipid species, sphingolipids were most significantly changed in both cell lines overexpressing UGT2B17 (Figure 1A). The most affected sphingolipids were the glycosphingolipids lactosylceramides (LacCer) and hexosylceramides (HexCer). HexCer comprise metabolites that have a neutral sugar moiety linked to a ceramide and include glucosylceramides (GluCer) and galactosylceramides (GalCer), which are not distinguished by this assay. Heatmaps showed that the majority of LacCer levels were significantly lower by 0.43‐ and 0.72‐fold in JVM2 and MEC1, respectively (*p* ≤ .0003), along with reduced sphingomyelins (C14:0, C16:0 and C18:1) (Figure 1B and Supporting Information Table S3). In contrast, HexCer (C16:0, C18:0, C20:0 and C22:0) and ceramides (Cer) (C20:0, C22:0, C24:0, C24:1 and C26:1) were increased. Additional components of the De novo pathway were decreased including sphinganine (−0.43‐fold; *p* < .0001) and palmitate (−0.35‐fold; *p* = .03), in parallel with higher coenzyme A levels (2.49‐fold; *p* = .013) (Figure 1C). In support of an increased production of HexCer, cellular levels of uridine diphosphate‐glucose (UDP‐glucose, −0.53‐fold, *p* = .001) and UDP‐galactose (−0.61‐fold, *p* = .009) were significantly depleted. UDP‐glucose and UDP‐galactose are required for the biotransformation of ceramide by glycosyltransferases UGCG and UGT8 to produce GluCer and GalCer, respectively.

We then carried out targeted lipidomics to quantify sphingolipid species, including Cer and GluCer, with a specific focus on the fatty‐acid chains C16:0 and C24:1, as well as sphingosine, dihydrosphingosine (also termed sphinganine) and sphingosine‐1‐phosphate (S1P) in three CLL cell lines with low and high expression of the adverse marker UGT2B17. Notably, high UGT2B17 expression was associated with enriched intracellular levels of C16:0 GluCer and C24:1 GluCer, as compared with controls for all three cell lines (Figure 1D). Altogether, the results are consistent with extensive metabolome perturbations associated with UGT2B17 in leukaemic cells as characterized by an enhanced production of HexCer and, specifically, GluCer (Figure 1B‐E), implying increased glycosylation pathways.

### 3.2 Elevated expression of GluCer‐producing pathways is a feature of CLL associated with poor survival

**FIGURE 2:**
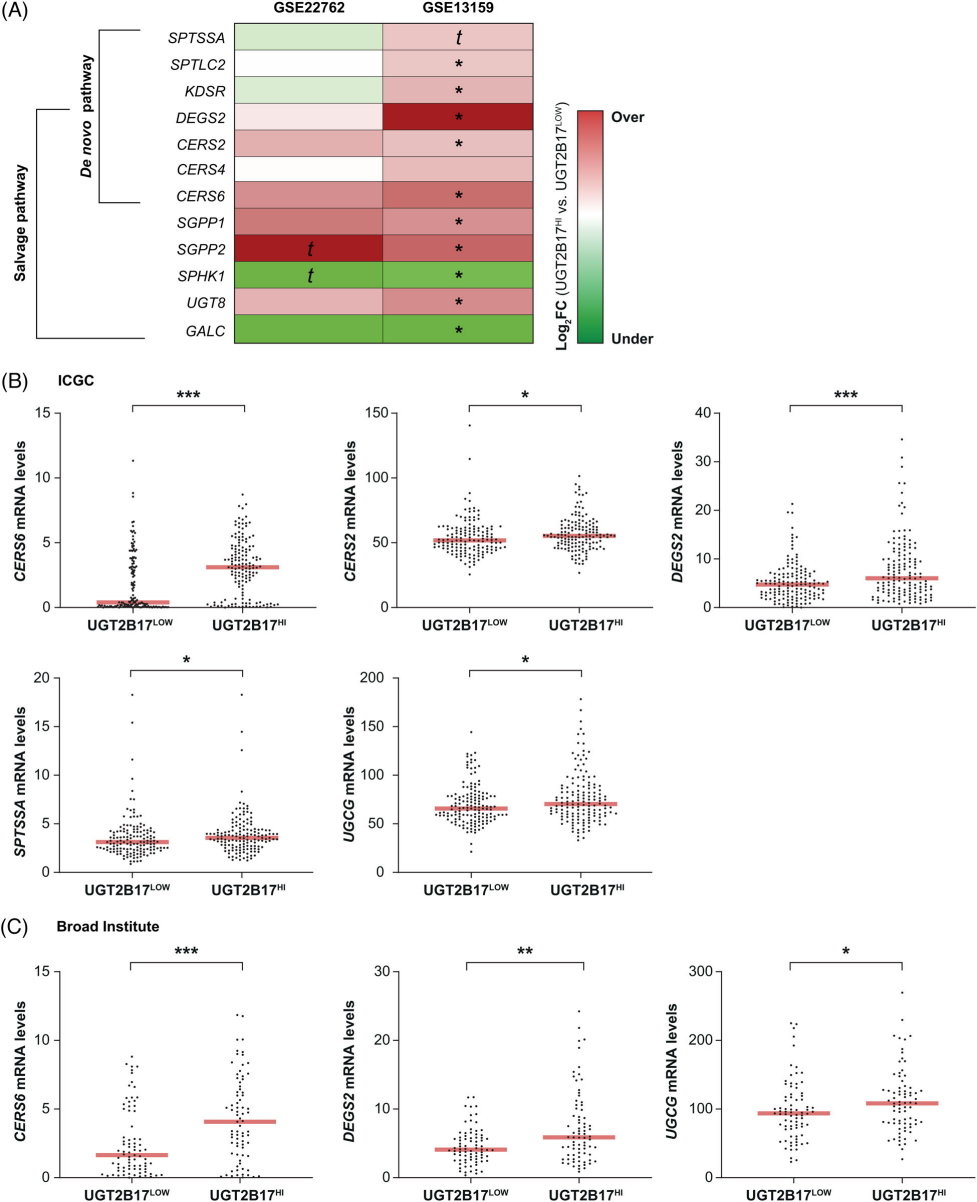
High *UGT2B17* expression is linked to enhanced expression of several sphingolipid biosynthetic pathways in independent cohorts of CLL patients. (A‐C) Gene expression of sphingolipid pathways was increased in UGT2B17^HI^ cases as compared with UGT2B17^LOW^ cases in four public datasets GSE22762 (n = 151) and GSE13159 (n = 448) (A), in the ICGC cohort comprising 294 cases (B) and the Broad Institute cohort with 156 cases (C). Genes for which mRNA levels were significantly altered by *UGT2B17* expression in CLL patients were included in (B‐C). Median *UGT2B17* expression levels (red line) were used to stratify patients. Scaled expression colour in (A) is related to the maximum (red, overexpression) and minimum (green, underexpression) value of log_2_ (fold change). *t* (trend): .05 ≤ *p* < .10; *p < .05; **p < .01; ***p < .001. *SPHK1*: sphingosine kinase 1.

We next evaluated whether the expression of enzymes and receptors involved in key pathways for sphingolipid production and action was changed in CLL cells with high *UGT2B17* expression. This was studied in four CLL cohorts, namely GSE22762 and GSE13159 (Figure 2A), ICGC (Figure 2B) and Broad Institute (Figure 2C), which have 151, 448, 294 and 156 cases, respectively. Levels of genes that encode enzymes of HexCer‐producing pathways, namely *UGT8* and *UGCG*, which are responsible for GalCer and GluCer synthesis, respectively, were significantly higher in cases with elevated expression of the adverse *UGT2B17* marker (i.e., UGT2B17^HI^) (Figure 2A‐C). In contrast, lower *GALC* expression, encoding the protein responsible for the hydrolysis of GalCer, was observed (*p* < .05; Figure 2A). Additional pathways involved in sphingolipid production, such as ceramide synthases *CERS2* and *CERS6*, delta 4‐desaturase sphingolipid 2 (*DEGS2*) and serine palmitoyltransferase small subunit A (*SPTSSA*), as well as serine palmitoyltransferase long‐chain base subunit 2 (*SPTLC2)*, 3‐ketodihydrosphingosine reductase (*KDSR)* and sphingosine‐1‐phosphate phosphatase (*SGPP1* and *SGPP2*), were significantly elevated in UGT2B17^HI^ patients as compared with UGT2B17^LOW^ cases (Figure 2A‐C). These observations support an enhanced synthesis of GluCer associated with overexpression of the adverse *UGT2B17* marker in CLL cases.

**FIGURE 3:**
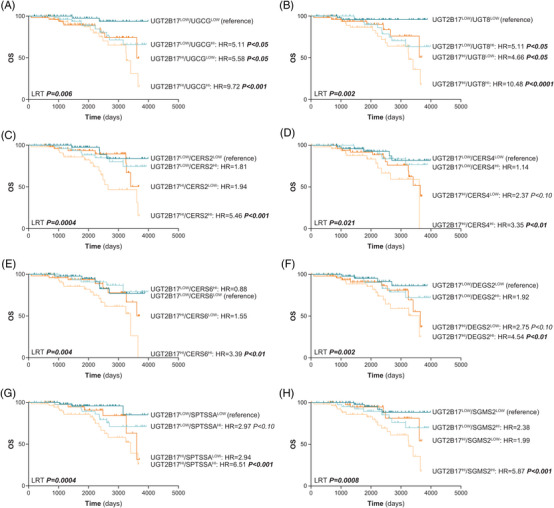
Association between *UGT2B17* expression in combination with ceramide‐ and hexosylceramide‐producing pathways and overall survival within a CLL cohort. (A‐F) Kaplan–Meier survival analyses for the combined expression of *UGT2B17* and genes that encode enzymes in the hexosylceramide‐producing (A‐B) and ceramide‐producing (C‐F) pathways in 191 CLL patients of the ICGC cohort. Expression levels were dichotomized based on median expression levels. Significant *p*‐values (<.05), including those from the log‐rank test (LRT), are highlighted in bold. Hazard ratios (HR) are shown. OS, overall survival.

A potential association with aggressiveness of the disease was further investigated in Kaplan–Meier survival analyses. We initially tested whether leukaemic cell expression of sphingolipid biosynthetic pathways was linked to OS in the ICGC cohort of 294 patients that consisted of treatment‐naïve cases. Sphingolipid pathways are complex and involve numerous enzymes. *De novo* synthesis of ceramides starts with the condensation of serine and palmitoyl‐CoA to produce 3‐ketosphinganine (Supporting Information Figure S1). Ceramides can also be generated by recycling sphingosine and HexCer in the salvage pathway and by hydrolysis of sphingomyelins. Overexpression of 11 of 59 investigated genes (19%) which encode enzymes and receptors involved in these pathways, was significantly associated with poor OS, supporting the potential roles of sphingolipids in leukaemic progression, both in males and females (Supporting Information Tables S4‐S6). Higher expression of the HexCer‐producing enzymes *UGCG* and *UGT8* was associated with poor OS (hazard ratio [HR] values of 2.03 and 2.69, respectively; *p* ≤ .02) (Supporting Information Figure S2A). In addition, high expression of several genes implicated in ceramide production such as *SPTSSA*, *DEGS2*, and multiple ceramide synthases *(CERS2*, *CERS4* and *CERS6)* was associated with HR values ranging from 1.99 to 4.41 (*p* ≤ .03) (Supporting Information Figure S2B). We further extended the analysis to the stratification of patients based on combined expression of *UGT2B17* and *UGCG*, which revealed inferior outcome in UGT2B17^HI^ and UGCG^HI^ cases (HR = 9.72, *p* < .001) as compared with UGT2B17^LOW^ and UGCG^LOW^ individuals (Figure 3A). We found similar results in the combined analysis of UGT2B17 and UGT8, enzymes involved in ceramide homeostasis *(CERS2*, *CERS4*, *CERS6*, *DEGS2, SPTSSA* and *SGMS2)* (Figure 3B‐H). These findings expose the clinical relevance of deregulated Cer and HexCer biosynthesis pathways in CLL progression and suggest a combined influence of the UGT2B17 metabolic pathway and the enzymes involved in the sphingolipid synthesis pathways such as UGCG.

### 3.3 Sphingolipids are upregulated in blood samples of CLL cases and are associated with aggressive disease and poor survival

**FIGURE 4:**
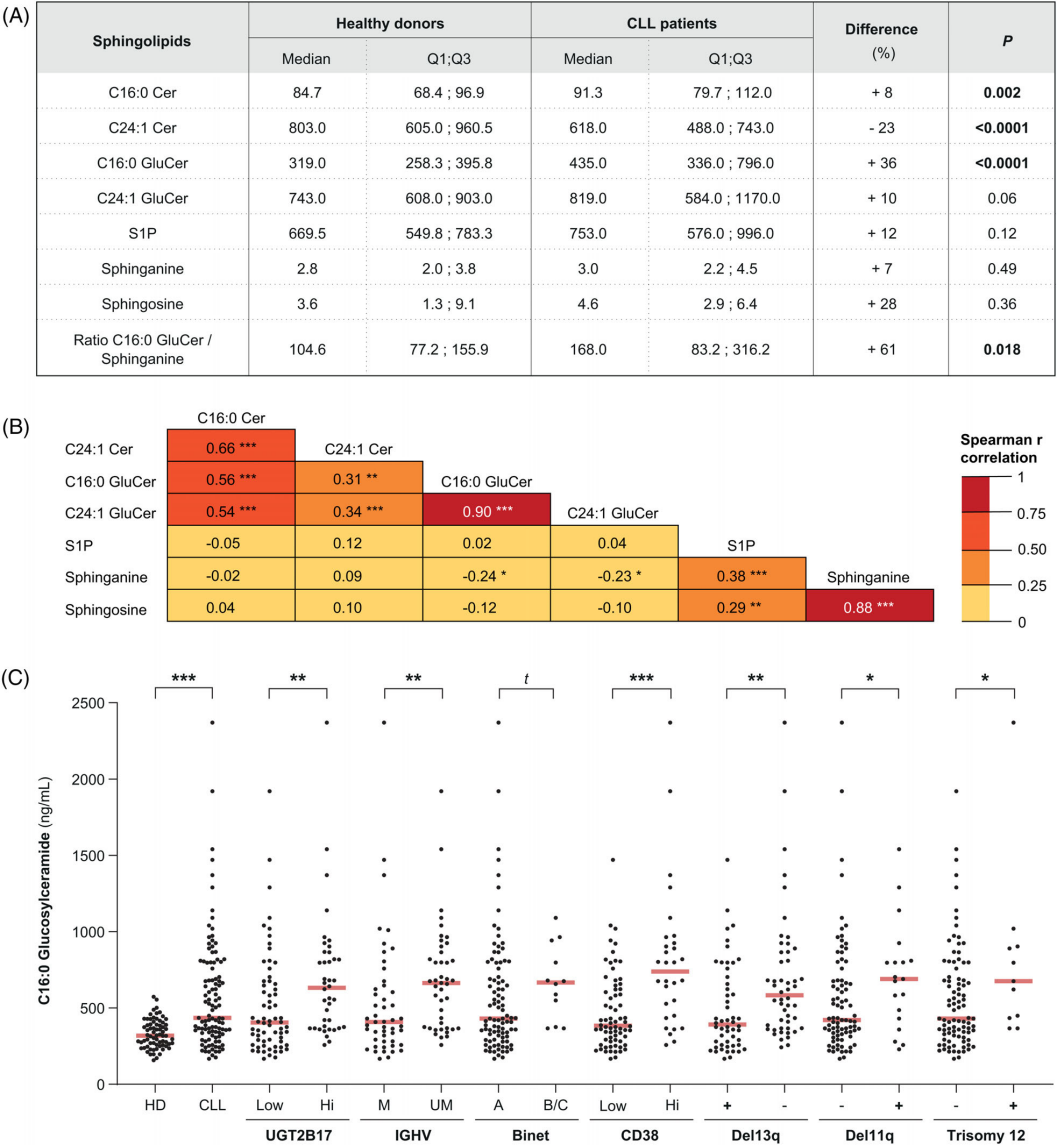
Circulating levels of sphingolipids, their correlation and changes according to adverse clinical, cytogenetic and molecular features in CLL patients. (A) Comparison of circulating sphingolipid levels measured in plasma samples of 107 CLL cases and 70 healthy donors.Transition and energies for each sphingolipid measured by mass spectrometry are provided in Supporting Information Table S8. Data are expressed as the median and first (Q1) and third (Q3) quartiles. Differences between cases and healthy donors are expressed as percentages (%) by comparing median values. The recovery of sphinganine and sphingosine‐1‐phosphate (S1P) was inferior in some serum samples available from healthy donors, and therefore, our analyses were restricted to plasma samples from 24 healthy donors. Significant differences (*p* < .05) are highlighted in bold. (B) Spearman r values for plasma sphingolipids measured in CLL patients. (C) Comparisons of median levels (red line) of circulating C16:0 GluCer between cases (CLL; n = 107) and healthy donors (HD; n = 70) and according to adverse features including *UGT2B17* expression levels measured in leukemic B cells (66 UGT2B17^HI^ vs. 40 UGT2B17^LOW^), IGHV mutation status (49 M‐CLL vs. 43 UM‐CLL), Binet stage A (n = 92) vs. stage B/C (n = 12), CD38 expression levels (40 CD38^HI^ vs. 70 CD38^LOW^), 13q deletion status (50 cases with no Del13q vs. 52 cases with Del13q), 11q deletion status (19 Del11q cases vs. 83 cases with no Del11q); trisomy 12 (11 positive cases vs. 91 negative cases). No significant difference in C16:0 GluCer was observed between a few carriers of the Del17p (n = 7) compared to the remaining cases (n = 95) (data not shown). Detailed quantitative sphingolipidomics data for CLL patients and healthy donors are provided in Supporting Information Table S9. *t* (trend): .05 ≤ *p* < .10; *p < .05; **p < .01; ***p < .001.

To evaluate the potential of circulating sphingolipids as biomarkers, we quantified sphingolipid levels using a targeted quantitative metabolomic assay (Supporting Information Figures S3, S4) in a cohort of 107 CLL cases (diagnosed at Vienna General Hospital) and 70 healthy donors (Supporting Information Table S1). As compared with healthy donors, cases displayed significantly higher circulating levels of C16:0 GluCer (Figure 4A). In contrast, lower levels of C24:1 Cer were observed, suggesting a redirection of sphingolipid metabolism toward GluCer production. No significant changes in sphinganine, sphingosine or S1P levels were noted (Figure 4A). There were moderate to strong correlations between circulating levels of Cer species (*r* = .66, *p* < .0001) and of GluCer species (*r* = .90, *p* < .0001), as well as between sphinganine and sphingosine levels (*r* = .88, *p* < .0001) (Figure 4B).

Upon stratification for high‐risk features such as IGHV mutational status, UM‐CLL had significantly higher C16:0 GluCer (76%; *p* = .0001; Figure 4C) and C24:1 GluCer (33%; *p =* .003, data not shown) with no significant changes in C16:0 Cer and C24:1 Cer levels or in sphinganine and sphingosine levels, relative to M‐CLL, which have a more favourable prognosis (data not shown). Consistent with our observations in cell models, UGT2B17^HI^ cases, which have more aggressive disease,^21^, ^22^, ^23^ had significantly higher circulating concentrations of C16:0 GluCer (56%; *p =* .002; Figure 4C) and, to a lesser extent, C24:1 GluCer (31%; *p =* .019; data not shown), as compared with UGT2B17^LOW^ cases. This was consistent with the analysis of B‐CLL cells from ten UGT2B17^HI^ cases that had a 1.3‐fold higher GluCer/Cer ratio that, however, did not reach significance, as compared to nine UGT2B17^LOW^ cases (data not shown). No significant changes in Cer, sphinganine, sphingosine and S1P levels were observed between UGT2B17^HI^ and UGT2B17^LOW^ patients. Higher levels of C16:0 GluCer were further noted in relation to additional high‐risk features including Binet stages B/C (55%; *p* = .058), high CD38 expression (92%; *p* < .0001), absence of 13q deletion (49%; *p* = .003), presence of 11q deletion (64%; *p* = .045) and presence of trisomy 12 (57%, *p* = .049) (Figure 4C). No significant sex differences were noted, except for circulating levels of sphinganine, which were higher in female patients (38%; *p* = .026) as compared with male patients (data not shown). These observations demonstrate increased circulating GluCer levels in CLL cases, especially in those with characteristics associated with a more aggressive disease.

**FIGURE 5:**
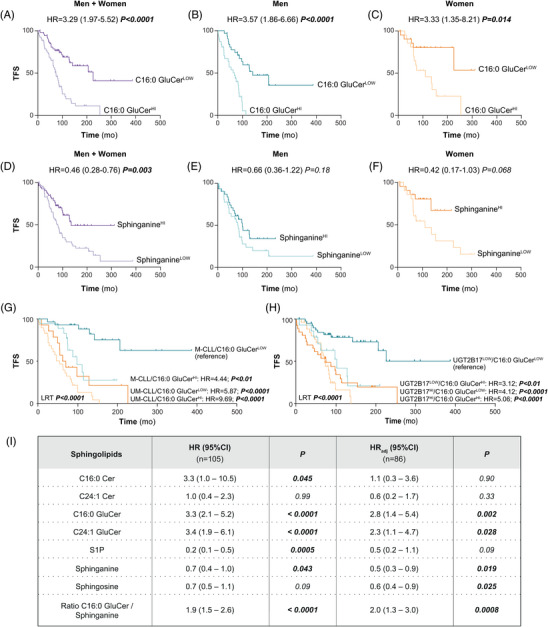
Survival analyses in relation to circulating sphingolipid levels in CLL patients. (A‐F) Kaplan–Meier analyses were performed for C16:0 GluCer (A‐C) and sphinganine (D‐F) levels in conjunction with treatment‐free survival (TFS) in a cohort of 107 CLL cases (A, D) comprising 63 men (B, E) and 44 women (C, F). Kaplan–Meier analyses for C16:0 GluCer with TFS were realized in combination with IGHV mutation status (G) and *UGT2B17* expression (H). Median levels were used to categorize cases according to high and low levels of sphingolipids. Hazard ratio (HR) values and 95% confidence intervals (95%CI) were calculated. (I) Multivariable Cox models were modelled without (HR) and with (HRadj) adjustment for CD38 expression, Binet staging, 11q deletion status and IGHV mutation status. A Cox model that also included UGT2B17 as a covariable provided similar results (data not shown). Significant *p*‐values (< .05) are highlighted in bold. Mo, months; LRT, log‐rank test.

In a Kaplan–Meier survival analysis of the same population of 107 CLL cases, higher circulating levels of C16:0 GluCer and C24:1 GluCer were significantly associated with shorter TFS (Figure 5A and Supporting Information Figure S5C). The association between C16:0 GluCer and TFS remained significant for men and women (Figure 5B,C). An HR value of 1.68 (95% CI = 1.01−2.80; *p* = .039) was also observed for levels of C16:0 Cer in all cases (Supporting Information Figure S5A). In contrast, higher circulating sphinganine levels were significantly associated with improved TFS (Figure 5D), a relationship that did not reach significance upon stratification by sex (Figure 5E,F).

The combination of C16:0 GluCer and IGHV mutation status resulted in significant outcome differences between groups (LRT *p* < .0001), supporting the potential use of ceramides to refine risk classification (Figure 5G). The same was observed for C16:0 GluCer in combination with UGT2B17 expression (Figure 5H). This was confirmed in multivariate analyses showing that both C16:0 GluCer and sphinganine were independently associated with TFS, with HR_adj._ values of 2.78 (1.44−5.38; *p* = .002) and .51 (.29−.89; *p* = .019), respectively (Figure 5I). Lastly, in the analysis of the C16:0 GluCer/sphinganine ratio, an increased risk of poor TFS was observed (HR_adj_ = 1.97; 95% CI = 1.96−2.59; *p* = .0008), supporting a predominant effect of the C16:0 GluCer metabolite on survival outcome (Figure 5I). Overall, data indicate that circulating sphingolipids are generally elevated in CLL cases compared to healthy donors, whereas C16:0 GluCer and sphinganine are specifically associated with patients’ survival.

### 3.4 Sphingolipids affect the proliferation and survival of leukaemic B cells

**FIGURE 6:**
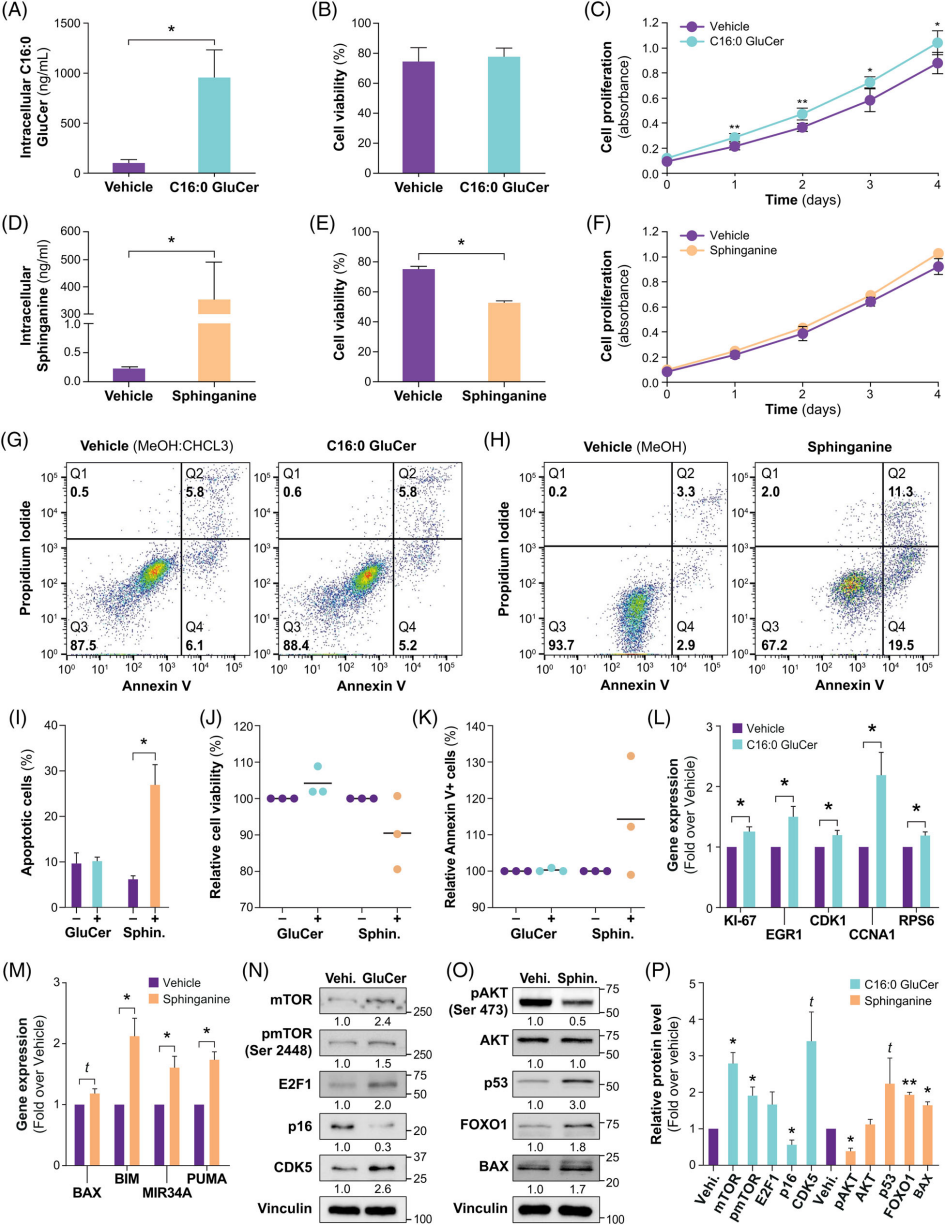
Treatment of leukemic cells with sphingolipids leads to altered proliferation and survival. (A–I, L–O) JVM2 cells were treated with 10 μM of C16:0 glucosylceramide (C16:0 GluCer) or 1:1 MeOH/CHCL_3_ (vehicle; A–C, G, I, L, N) or 10 μM sphinganine or MeOH (vehicle; D–F, H–I, M, O). Treatment efficacy was confirmed by MS‐based measures of intracellular C16:0 GluCer (A) and sphinganine (D). Cell viability (B, E), cell proliferation (C, F) and apoptosis (G–I) were then assessed. Primary cells from CLL patients (n = 3) were treated with 10 μM C16:0 GluCer for 22 h or 10 μM sphinganine for 4 h or with their respective vehicle (J–K). Cell viability (J) and apoptosis (K) were assessed and reported relative to vehicle. Gene expression (L–M) and protein expression (N–O) analyses of selected candidates were carried out after treatment with C16:0 GluCer or sphinganine on JVM2 cells and are shown as fold change relative to vehicle. A representative experiment is shown and quantification for biological replicates are displayed in (P). Candidates from mTOR signalling pathways, involved in cell cycle regulation (L, N) and apoptosis regulation (M, O) were assessed. Data are expressed as the mean ± standard error from at least two independent experiments. Similar observations using a second cell model are displayed in Supporting Information Figure S6. Primers sequences used for gene expression quantification are listed in Supporting Information Table S10. *t* (trend): .05 ≤ *p* < .10; *p < .05; **p < .01; ***p < .001. Vehi., vehicle; Sphinga., sphinganine.

The potential mechanisms underlying these associations were investigated. Leukaemic cells treated with C16:0 GluCer for 48 h showed a significant ninefold increase in intracellular C16:0 GluCer relative to vehicle (*p* = .03) (Figure 6A; JVM2). These C16:0 GluCer experiments were challenging because of the poor solubility of this sphingolipid caused by the hydrophobic features of its long fatty acyl chain length.^28^ Under these treatment conditions, there was no effect on cell viability (Figure 6B,G,I) but a significant increase in cell proliferation was observed (by an average of 26%; *p* < .05) (Figure 6C). By contrast, sphinganine treatment led to a significant >100‐fold increase in intracellular sphinganine (*p* = .03) (Figure 6D), accompanied by a 27% reduction in cell viability (*p* = .02, Figure 6E) and a 27% increase in cell apoptosis based on flow cytometry analysis (*p* = .03) but no effect on cell proliferation (Figure 6F,H,I). These observations were replicated in a second leukaemic B‐cell model (HG3) (Supporting Information Figure S6A‐G). The initial findings in primary CLL cells from patients indicated a minor increase in cell viability, with no noticeable impact on apoptosis for C16:0 GluCer, while sphinganine increased cell apoptosis (Figure 6J‐K). Our findings, thus, support different biological functions in leukaemic B cells for C16:0 GluCer and sphinganine.

The pro‐proliferative effects of C16:0 GluCer and its effect as a modulator of gene expression were consistent with the upregulation of several markers at the mRNA level such as the marker of proliferation *KI‐67* and cell cycle‐related genes such as cyclin‐dependent kinases *(CDK1, CDK4)*, cyclins *(CCNA1, CCND1)* and early growth response 1 (*p* < .05) in both cell lines (Figure 6L and Supporting Information Figure S6H). At the protein level, cell cycle regulators p16 and E2F transcription factor 1 (E2F1) were also affected in both JVM2 and HG3 cell models (Figure 6N,P and Supporting Information Figure S6J,L). Evidence also points to the upregulation, at the protein level, of translational regulation driven by mTOR signalling, and ribosomal protein S6 at the mRNA level in cells treated with C16:0 GluCer (Figure 6L,N,P). The pro‐survival effect of sphinganine was consistent with the upregulation at mRNA levels of pro‐apoptotic factors, such as B‐cell CLL/lymphoma 2 (BCL2)‐interacting mediator of cell death (*BIM*), p53‐upregulated modulator of apoptosis (*PUMA)* and microRNA 34a (*MIR34A)* (*p* < .05) (Figure 6M) and higher protein expression of p53, forkhead box O1 (FOXO1) and BCL2‐associated *X*, apoptosis regulator (BAX), involved in apoptosis (Figure 6O,P). Similar findings were observed in a second CLL cell model (Supporting Information Figure S6H‐L).

**FIGURE 7:**
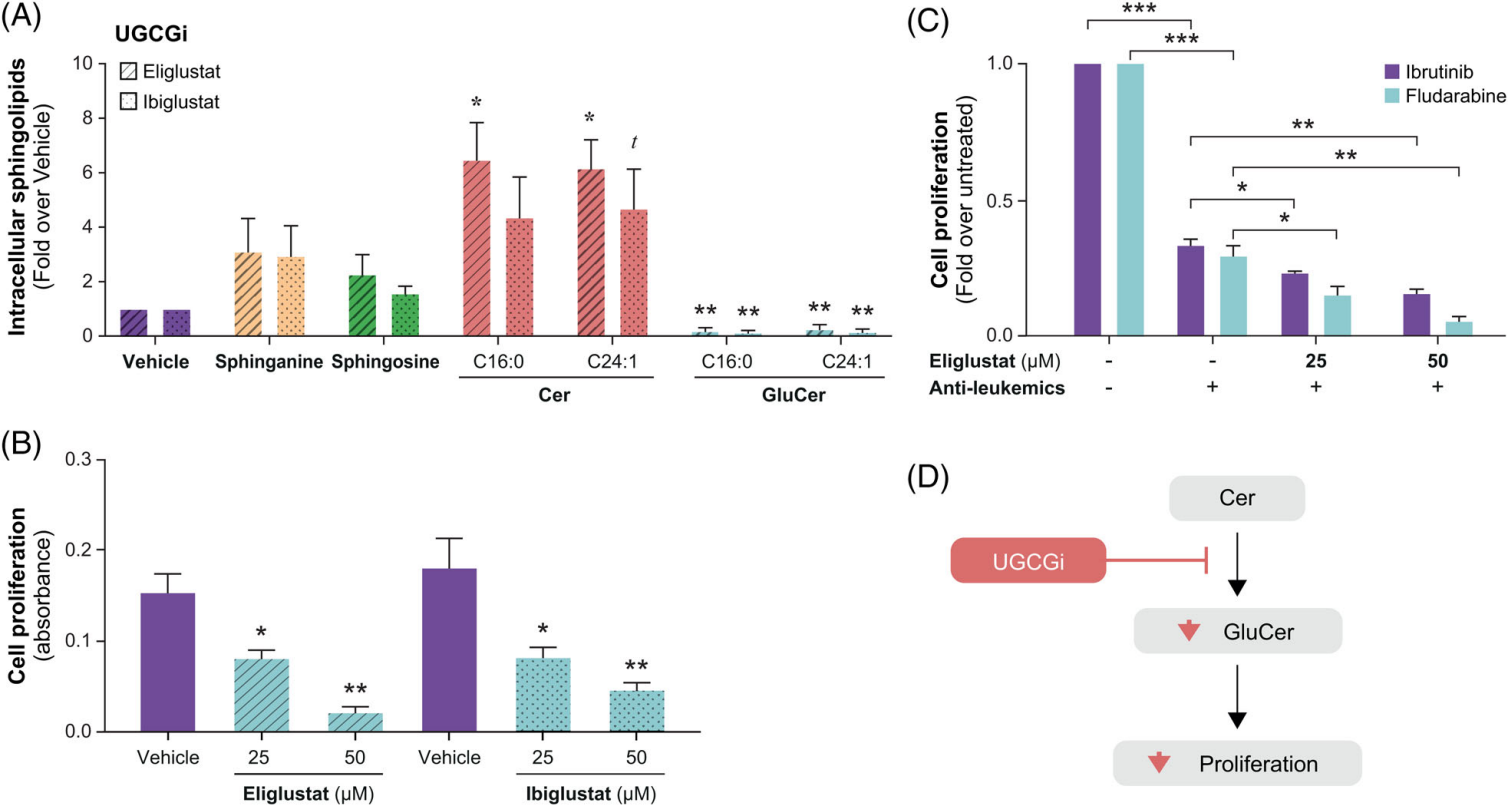
Treatment of leukemic cells with UGCG inhibitors (UGCGi) decreases the pro‐proliferative phenotype induced by C16:0 GluCer and enhances the effects of anti‐leukaemics. (A) Blockade of glucosylceramide synthesis was evaluated using two UGCGi, eliglustat and ibiglustat at 25 μM for 4 days, as compared with vehicle (DMSO). The efficacy and specificity of pharmacological inhibition of UGCG, responsible for the conversion of ceramides (Cer) to glucosylceramides (GluCer), was confirmed by MS‐based measures of intracellular GluCer. (B) Inhibition of cell proliferation after treatment with UGCGi at 25 and 50 μM for 4 days was compared with vehicle (DMSO). (C) Inhibition of cell proliferation using the anti‐leukaemics fludarabine (10 μM) and ibrutinib (1 μM) alone and in combination with 25 and 50 μM eliglustat treatment for 4 days. (D) Our data suggest that UGCG inhibition leads to the reduced conversion of Cer to GluCer in leukemic cells, strongly inhibiting cell proliferation. Data are expressed as the mean ± standard error from two independent experiments performed in JVM2 cells. *t* (trend): .05 ≤ *p* < .10; *p < .05; **p < .01; ***p < .001.

Blocking GluCer production with UGCGi was effective in reducing intracellular C16:0 GluCer (by 5.3‐fold relative to vehicle with eliglustat, *p* = .003; by 7.7‐fold relative to vehicle with ibiglustat, *p* = .003) and C24:1 GluCer (by fourfold with eliglustat, *p* = .008; by 6.3‐fold with ibiglustat, *p* = .004) (Figure 7A) and led to reduced cell proliferation of JVM2 cells (Figure 7B). This was also the case when UGCGi was combined with standard CLL treatments, such as ibrutinib and fludarabine, displaying decreases by 1.6‐fold (*p* = .003) and 2.6‐fold (*p* = .01), respectively (Figure 7C,D). Reduced cell viability was further associated with increased apoptosis for both ibrutinib (by 3.8‐fold, *p* = .015) and fludarabine (by 2.1‐fold, *p* = .017) when combined with 50 μM UGCGi (Supporting Information Figure S7). These results highlight the potential for UGCG‐based inhibition to reduce the production of pro‐proliferative GluCer.

## 4 DISCUSSION

Here, we assessed the altered metabolism of aggressive CLL by initially focusing on B cells that overexpress the metabolic marker UGT2B17, which is characteristic of a more aggressive disease and associated with poor survival outcomes.^21^, ^22^, ^23^ Using untargeted metabolomics and lipidomics, we uncovered significant perturbations of lipids, predominantly bio‐active sphingolipids, which are involved in a variety of cellular processes.^29^ More specifically, we found higher levels of intracellular HexCer including GluCer also linked to higher UGT2B17 leukaemic cell expression and increased levels of GluCer in B‐CLL cell models. Data implies increased glycosylation pathways supported by deregulated sphingolipid biosynthesis pathways associated with shorter survival of CLL patients. GluCer accumulation in indolent and aggressive cases suggests the potential involvement of this class of sphingolipids in CLL early‐stage pathogenesis and their possible usefulness as diagnostic biomarkers. Levels of circulating sphingolipid levels were also greater in CLL cases with high‐risk features including higher leukaemic expression of the prognostic marker UGT2B17. Furthermore, our findings highlight the independent prognostic value of GluCer and sphinganine in treatment‐naïve CLL patients, revealing their significant association with TFS. These observations support that sphingolipids could be useful for improved stratification of CLL patients and data support their influence on behaviour of leukaemic cells in vitro.

A limited number of metabolomics studies have been conducted in the context of CLL (Supporting Information Table S7) and none reported the potential prognostic value of sphingolipids in CLL patients.^5^, ^17^, ^18^, ^19^, ^20^ These investigations were, however, restricted in sample size and compared only a limited number of CLL cases to healthy donors, with few indolent and aggressive CLL cases characterized based on their IGHV mutation status. Five studies have reported an alteration in sphingolipids, mostly C16:0 and C24:1 Cer and GluCer, in CLL, which is consistent with our findings (Supporting Information Table S7).^17^, ^18^, ^20^, ^30^, ^31^ For instance, Thurgood and collaborators^20^ found elevated levels of GluCer but reduced ceramides and lactosylceramides, with no changes in sphingomyelins, as measured in B‐CLL cells isolated from 20 individuals classified as either UM‐CLL or M‐CLL and as compared with six healthy controls. In the present study, higher circulating levels of C16:0 GluCer emerged as an independent prognostic marker in previously untreated CLL cases, whereas levels of one of the Cer precursors, sphinganine (also termed dihydrosphingosine), had the opposite effect. The influence of sphingolipids was further supported by significant associations between leukaemic cell expression of numerous sphingolipid biosynthetic pathways, measured in specimens collected in treatment‐naïve patients and OS in a second cohort. The treatment received by patients has a significant role in determining their OS rates but the details of the treatment were not available for analysis of this cohort. Although gene expression analyses may not necessarily provide a direct reflection of enzyme activity or dependencies on specific metabolic pathways, the analysis of the transcription profile for multiple lipid pathways points toward the accumulation of HexCer and GluCer, consistent with cell‐based metabolomics observations. This suggests that sphingolipid metabolic alterations are intrinsic to leukaemic B cells and that these changes are reflected at the systemic level in CLL patients. Few studies are also suggesting a potential effect of the microenvironment on cellular sphingolipid levels that warrant additional investigations.^17^, ^32^

Based on our evidence of their opposing effects in association with TFS in multivariate analyses, sphingolipid species may have antagonistic roles in CLL progression, with C16:0 GluCer promoting tumour aggressiveness leading to shorter survival and sphinganine acting as a protective molecule leading to enhanced survival. Accordingly, the balance between these two sphingolipid species has the potential to influence clinical outcomes. We further observed that the ratio of C16:0 GluCer to sphinganine was predictive of a shorter TFS. This suggests a predominant impact of GluCer on CLL progression, also consistent with significantly higher concentrations of C16:0 GluCer as compared with sphinganine in circulation of CLL patients (median levels of 493 nM as compared with 10 nM, respectively). The importance of the tight regulation of sphingolipid metabolism homeostasis in cancer development and progression has been well established.^33^, ^34^, ^35^, ^36^ For instance, the concept of the sphingolipid rheostat involving the modulation of opposing sphingolipid signalling pathways as major determinants of cell fate has been recognized.^35^, ^37^, ^38^, ^39^ However, little is known about the role of GluCer and sphinganine in CLL.

The antagonistic effects of C16:0 GluCer and sphinganine are also supported by cell‐based investigations, with sphinganine displaying a pro‐apoptotic effect and C16:0 GluCer being pro‐proliferative, an effect that was reversed by inhibiting GluCer synthesis using UGCGi. Furthermore, a greater reduction in cell proliferation was observed after co‐treatment with UGCGi and the anti‐leukaemics fludarabine and ibrutinib, supporting the potential relevance of targeting sphingolipid pathways in CLL to synergistically enhance clinical responses.^40^ We observed that elevated intracellular sphinganine promoted apoptosis, reduced the activation of the AKT signalling pathway and enhanced the expression of the transcription factors p53 and FOXO1 and their target genes.^41^, ^42^ Consistent with these findings, previous studies support the potential of sphinganine to induce apoptosis in colon cancer^43^, ^44^ and breast cancer^45^ cells. In contrast, treatment of leukaemic B cells with C16:0 GluCer led to increased cell proliferation. Our investigations also indicate that GluCer might be influencing p16 levels, which, in turn, could affect downstream targets, like mTOR pathway^46^ and E2F1,^47^, ^48^ leading to potential alterations in protein synthesis, cell cycle regulation and cell proliferation. Our observations are supported by the work of Huang and colleagues,^30^ who showed that treatment with various GluCer concentrations promotes cell proliferation of the MEC‐2 cell line, although they did not specify which GluCer species was (or were) used to treat the cells. Additional lines of evidence suggest that GluCer affects the proliferation potential of various cell types,^30^, ^49^ but the exact molecular mechanisms remain unknown and will need to be further investigated. Besides, studying sphingolipids presents significant challenges due to their unique characteristics and complex metabolism.^50^ The hydrophobic nature of sphingolipids makes challenging their delivery to cells, especially for complex sphingolipids such as GluCer. This may potentially explain the more subtle changes in cell phenotype induced by the supplementation of cells with C16:0 GluCer compared to sphinganine. In addition, the interconversion of sphingolipids by the cellular machinery is a complex process involving various enzymes and pathways, thus, making it difficult to ascertain the effect of a specific sphingolipid species. Future studies using specific inhibitors of enzymes involved in sphingolipid metabolism will help in dissecting their contributions. Thus, altering the levels of GluCer can have intricate and interconnected effects with Cer on cellular processes. For instance, ceramides are known to induce programmed cell death, and their conversion to GluCer would, therefore, prevent apoptosis.^17^, ^51^ Accordingly, nanoliposomal C6‐Cer is currently being tested as an anti‐tumorigenic agent for advanced solid tumours and acute myeloid leukaemia (NCT02834611; NCT04716452) and is being developed to overcome the cell impermeability of Cer and its precipitation in aqueous solution.^52^, ^53^ However, in our study, Cer levels remained unaffected in cell lines and CLL patients and were not significantly associated with TFS, further suggesting that our findings were likely triggered by changes in GluCer.

UGCG is upregulated under conditions that promote survival of primary CLL cells, such as B‐cell receptor engagement, leading to enhanced glycosylation of ceramides and further supporting a pro‐survival role for GluCer.^17^ Our experiments targeting the production of GluCer by inhibiting the rate‐limiting UGCG enzyme are promising, as a significant reduction in cell proliferation was observed upon UGCGi treatment, namely using the well‐tolerated eliglustat approved for Gaucher disease type 1.^54^, ^55^ Gaucher disease is a metabolic disease caused by a deficiency of the acid β‐glucosidase that converts GluCer into glucose and ceramide. This rare genetic disease is characterized by an accumulation of GluCer and is associated with an increased risk of hematologic malignancies including CLL.^56^ This suggests that GluCer may be involved in the development of CLL and is supported by our observations of higher levels of GluCer in CLL patients as compared with healthy donors. In addition, the inhibitory effect of UGCGi appears to sensitize cells to prevailing CLL treatments, such as ibrutinib and fludarabine in our investigations, suggesting that targeting sphingolipids may provide additional benefits. Our observations, thus, align with the findings previously reported in two studies, which demonstrate that treating cells with UGCGi significantly improves the treatment response to anti‐leukaemic in primary CLL cells. Schwamb et al.^17^ found that inhibition of UGCG may be efficient in modifying the ceramide and glucosylceramide equilibrium and restoring sensitivity to kinase inhibitors in primary CLL cells. In addition, alteration of sphingolipid metabolism leads to an accumulation of GluCer that is associated with fludarabine resistance and can be reversed by UGCG inhibition.^30^ Taken together, these data underscore the potential for UGCG‐based inhibition to reduce production of pro‐proliferative GluCer during the treatment of CLL.

## 5 CONCLUSIONS

Through metabolomics approaches, we have successfully pinpointed metabolic vulnerabilities linked to aggressive CLL, underscoring their promising applications as novel circulating biomarkers and potential therapeutic targets. Utilizing plasma samples to assess sphingolipid biomarkers presents several advantages, notably non‐invasiveness and ease of collection. Additionally, our initial mechanistic discoveries propose that the rewiring of sphingolipid metabolism may play a significant role in driving progressive disease via the accumulation of pro‐proliferative GluCer and the depletion of the anti‐apoptotic sphinganine. Additional studies are required to validate the multilevel involvement of sphingolipids and their prognostic value and to gain mechanistic knowledge about these altered sphingolipid pathways, which could then be leveraged into improved therapeutic strategies to treat CLL patients.

## AUTHOR CONTRIBUTIONS

Conceived and designed the study: CG. Recruited patients and acquired clinical data: KV. Conducted experiments and analyzed data: FNVL, DVG, PC, MR, LV, RS, TL, KV, CG. Performed statistical analyses: FNVL, DS. Drafted the manuscript: FNVL, CG. Critical revision of the manuscript for important intellectual content: All authors. Obtained funding: CG.

## FUNDING INFORMATION

This work was supported by research grants from the Canadian Institutes of Health Research (CIHR) (FRN‐167269 to CG) and the Canada Research Chair Program (CG). FNVL was awarded a post‐doctoral scholarship from Cancer Research Center (CRC) ‐ Université Laval. IL holds a clinical research scholar with funding from the Fonds de Recherche du Québec – Santé (FRQS). The project was also made possible with the support of the Canada Foundation for Innovation (John R. Evans Leaders Funds #34272 to CG and #37996 to IL). CG holds the Canada Research Chair in Pharmacogenomics (Tier I).

## CONFLICT OF INTEREST STATEMENT

The authors declare no competing financial interest.

## ETHICAL APPROVAL

The studies involving human participants were reviewed and approved by the Ethics committee of the CHUQc – Université Laval (#2015‐1205) and Medical University of Vienna (Ethics vote 2176/2017). The patients/participants provided their written informed consent to participate in this study.

## Supporting information

Supporting Information

Supporting Information

Supporting Information

Supporting Information

## Data Availability

The original contributions presented in the study are included in the article/supplementary material. Further inquiries can be directed to the corresponding authors.
