# Supplementary material for: Untargeted metabolomics identifies metabolic dysregulation of sphingolipids associated with aggressive chronic lymphocytic leukaemia and poor survival
Source: Clin Transl Med. 2023 Nov 30;13(12):e1442. doi: 10.1002/ctm2.1442 (PMC10689972; doi:10.1002/ctm2.1442)
Supplement: Supplementary file 1 — Supporting Information [file CTM2-13-e1442-s001.pptx]

## Slide 1
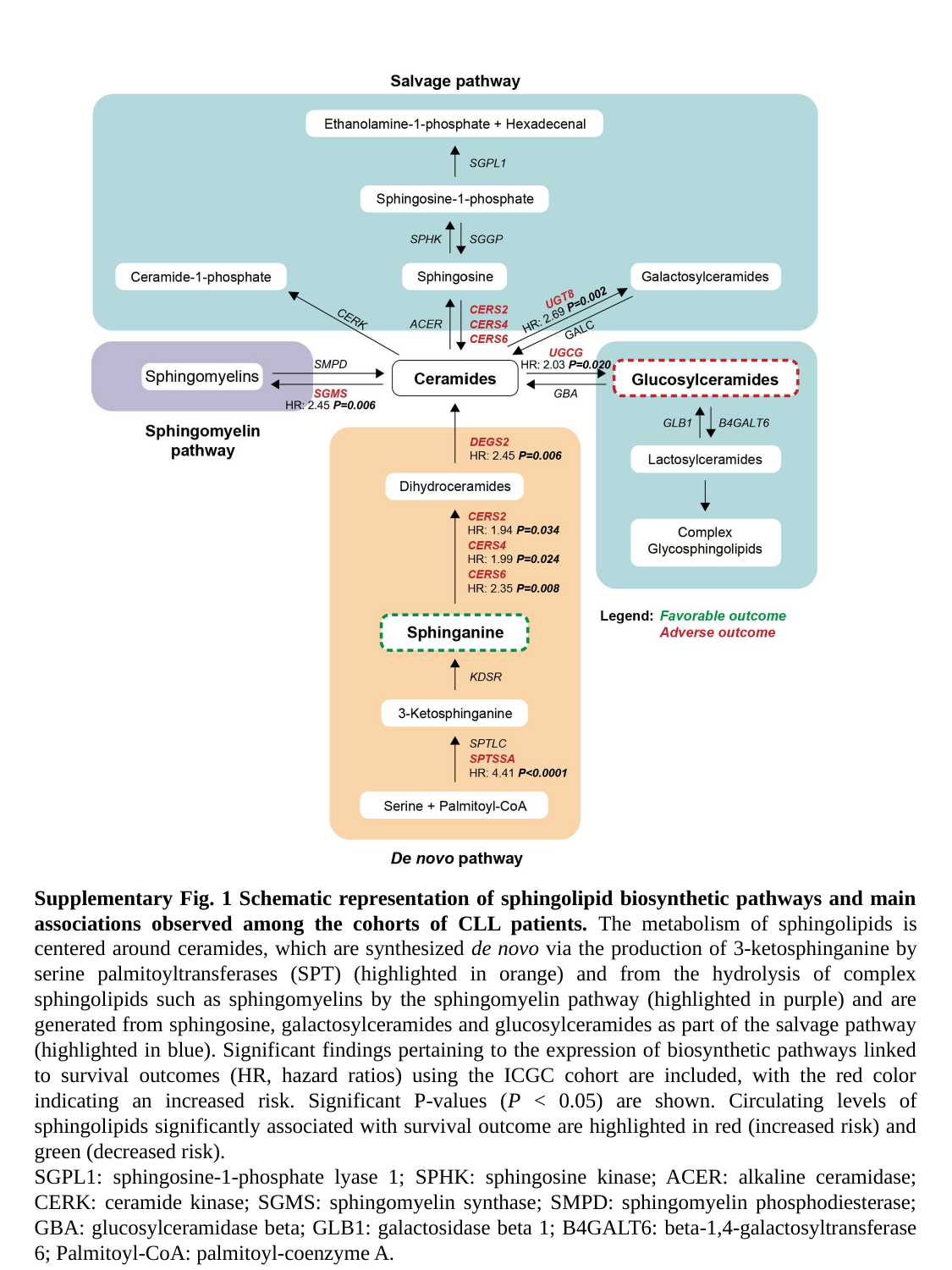

Supplementary Fig. 1 Schematic representation of sphingolipid biosynthetic pathways and main associations observed among the cohorts of CLL patients. The metabolism of sphingolipids is centered around ceramides, which are synthesized de novo via the production of 3-ketosphinganine by serine palmitoyltransferases (SPT) (highlighted in orange) and from the hydrolysis of complex sphingolipids such as sphingomyelins by the sphingomyelin pathway (highlighted in purple) and are generated from sphingosine, galactosylceramides and glucosylceramides as part of the salvage pathway (highlighted in blue). Significant findings pertaining to the expression of biosynthetic pathways linked to survival outcomes (HR, hazard ratios) using the ICGC cohort are included, with the red color indicating an increased risk. Significant P-values (P < 0.05) are shown. Circulating levels of sphingolipids significantly associated with survival outcome are highlighted in red (increased risk) and green (decreased risk).
SGPL1: sphingosine-1-phosphate lyase 1; SPHK: sphingosine kinase; ACER: alkaline ceramidase; CERK: ceramide kinase; SGMS: sphingomyelin synthase; SMPD: sphingomyelin phosphodiesterase; GBA: glucosylceramidase beta; GLB1: galactosidase beta 1; B4GALT6: beta-1,4-galactosyltransferase 6; Palmitoyl-CoA: palmitoyl-coenzyme A.

## Slide 2
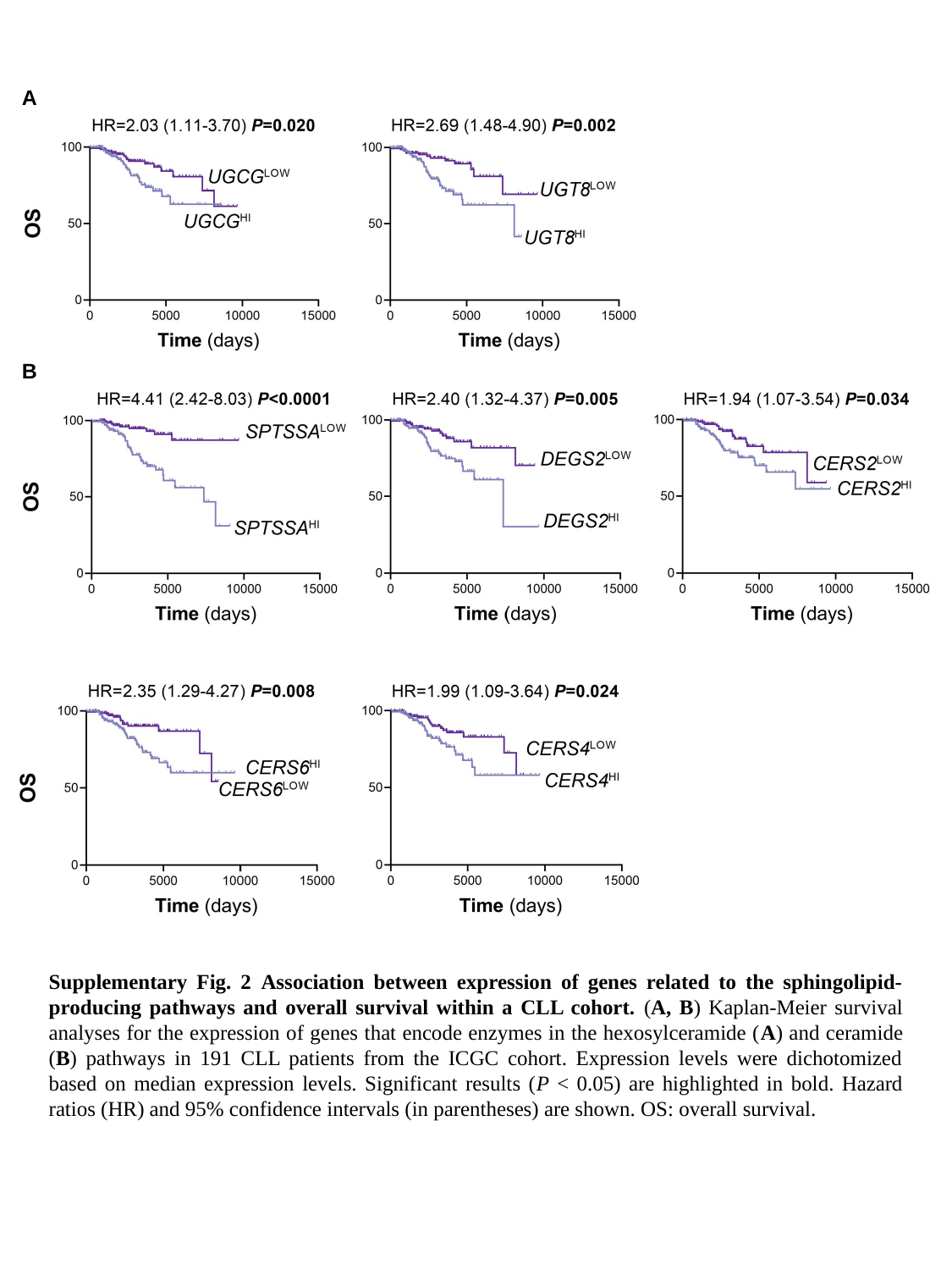

A
B
Supplementary Fig. 2 Association between expression of genes related to the sphingolipid-producing pathways and overall survival within a CLL cohort. (A, B) Kaplan-Meier survival analyses for the expression of genes that encode enzymes in the hexosylceramide (A) and ceramide (B) pathways in 191 CLL patients from the ICGC cohort. Expression levels were dichotomized based on median expression levels. Significant results (P < 0.05) are highlighted in bold. Hazard ratios (HR) and 95% confidence intervals (in parentheses) are shown. OS: overall survival.

## Slide 3
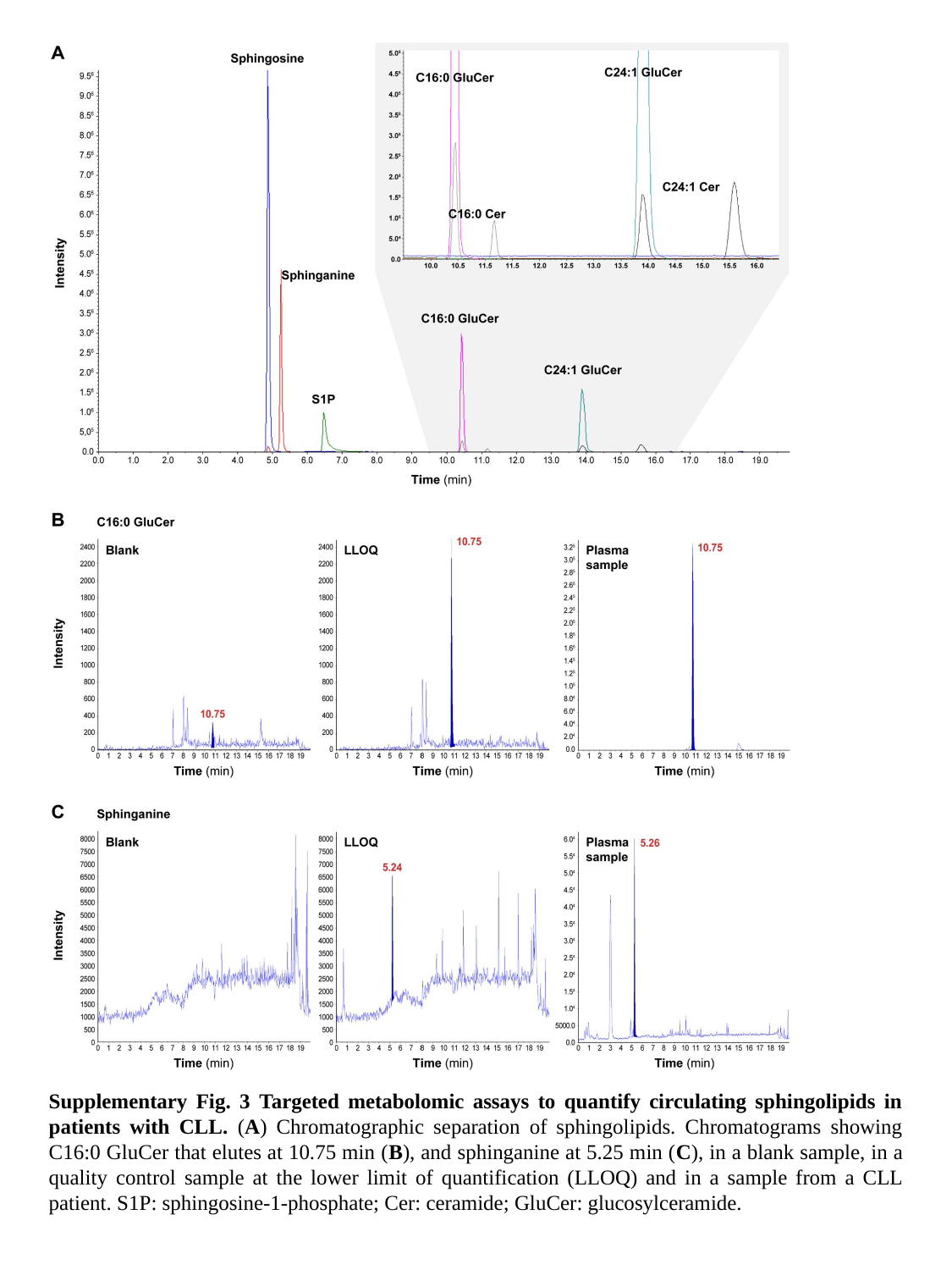

Supplementary Fig. 3 Targeted metabolomic assays to quantify circulating sphingolipids in patients with CLL. (A) Chromatographic separation of sphingolipids. Chromatograms showing C16:0 GluCer that elutes at 10.75 min (B), and sphinganine at 5.25 min (C), in a blank sample, in a quality control sample at the lower limit of quantification (LLOQ) and in a sample from a CLL patient. S1P: sphingosine-1-phosphate; Cer: ceramide; GluCer: glucosylceramide.

## Slide 4
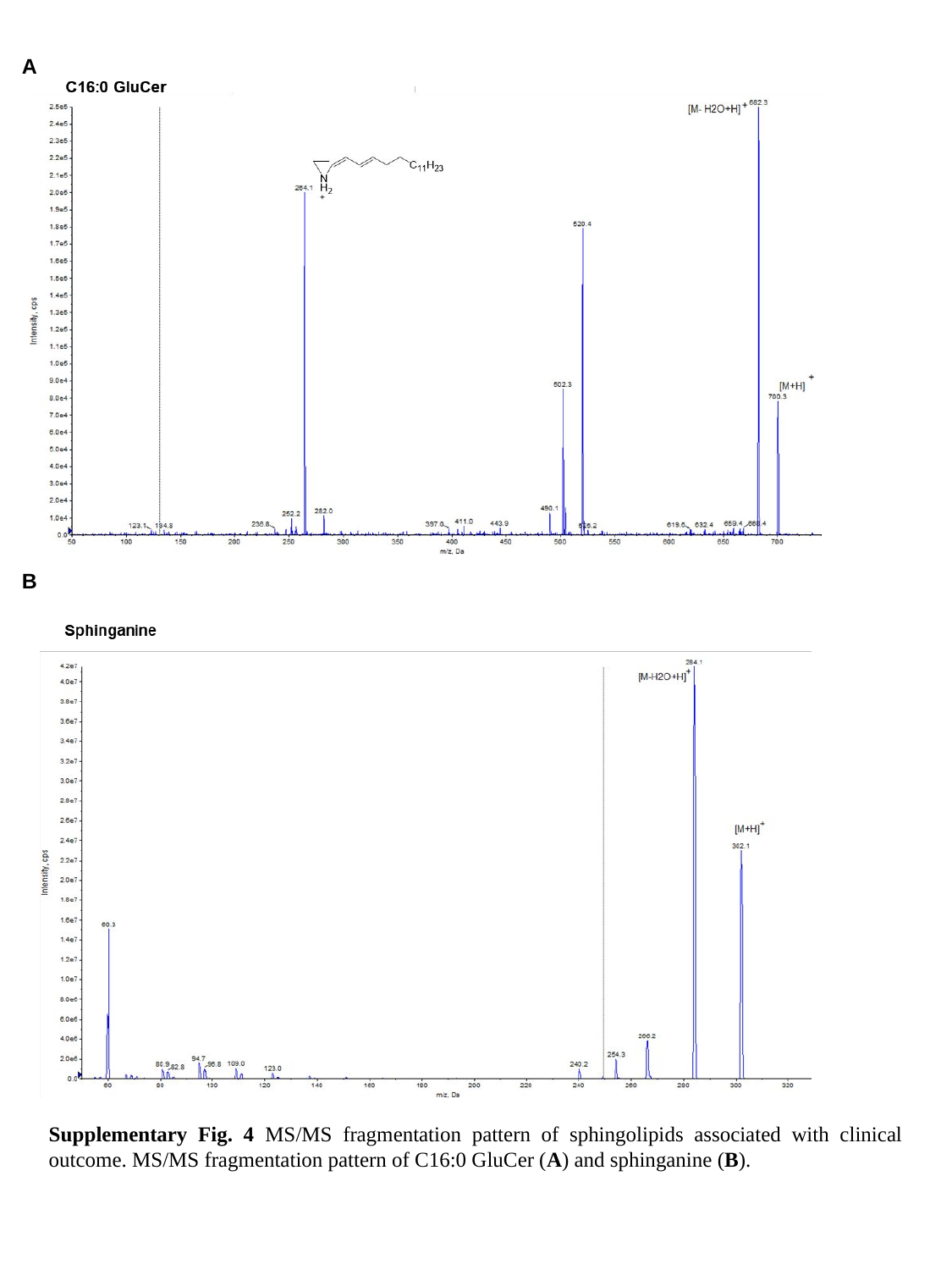

A
B
Supplementary Fig. 4 MS/MS fragmentation pattern of sphingolipids associated with clinical outcome. MS/MS fragmentation pattern of C16:0 GluCer (A) and sphinganine (B).

## Slide 5
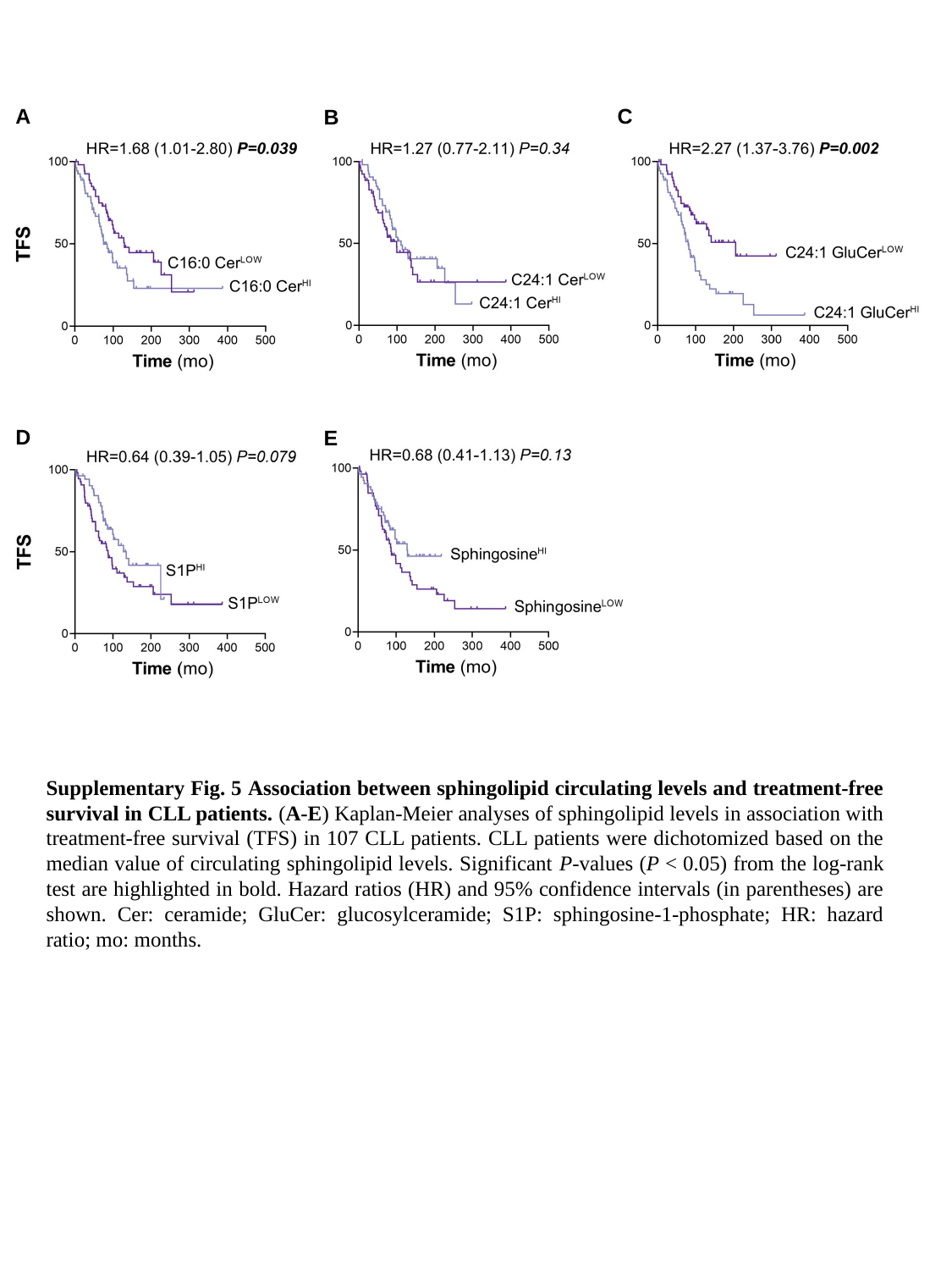

A
C
B
D
E
Supplementary Fig. 5 Association between sphingolipid circulating levels and treatment-free survival in CLL patients. (A-E) Kaplan-Meier analyses of sphingolipid levels in association with treatment-free survival (TFS) in 107 CLL patients. CLL patients were dichotomized based on the median value of circulating sphingolipid levels. Significant P-values (P < 0.05) from the log-rank test are highlighted in bold. Hazard ratios (HR) and 95% confidence intervals (in parentheses) are shown. Cer: ceramide; GluCer: glucosylceramide; S1P: sphingosine-1-phosphate; HR: hazard ratio; mo: months.

## Slide 6
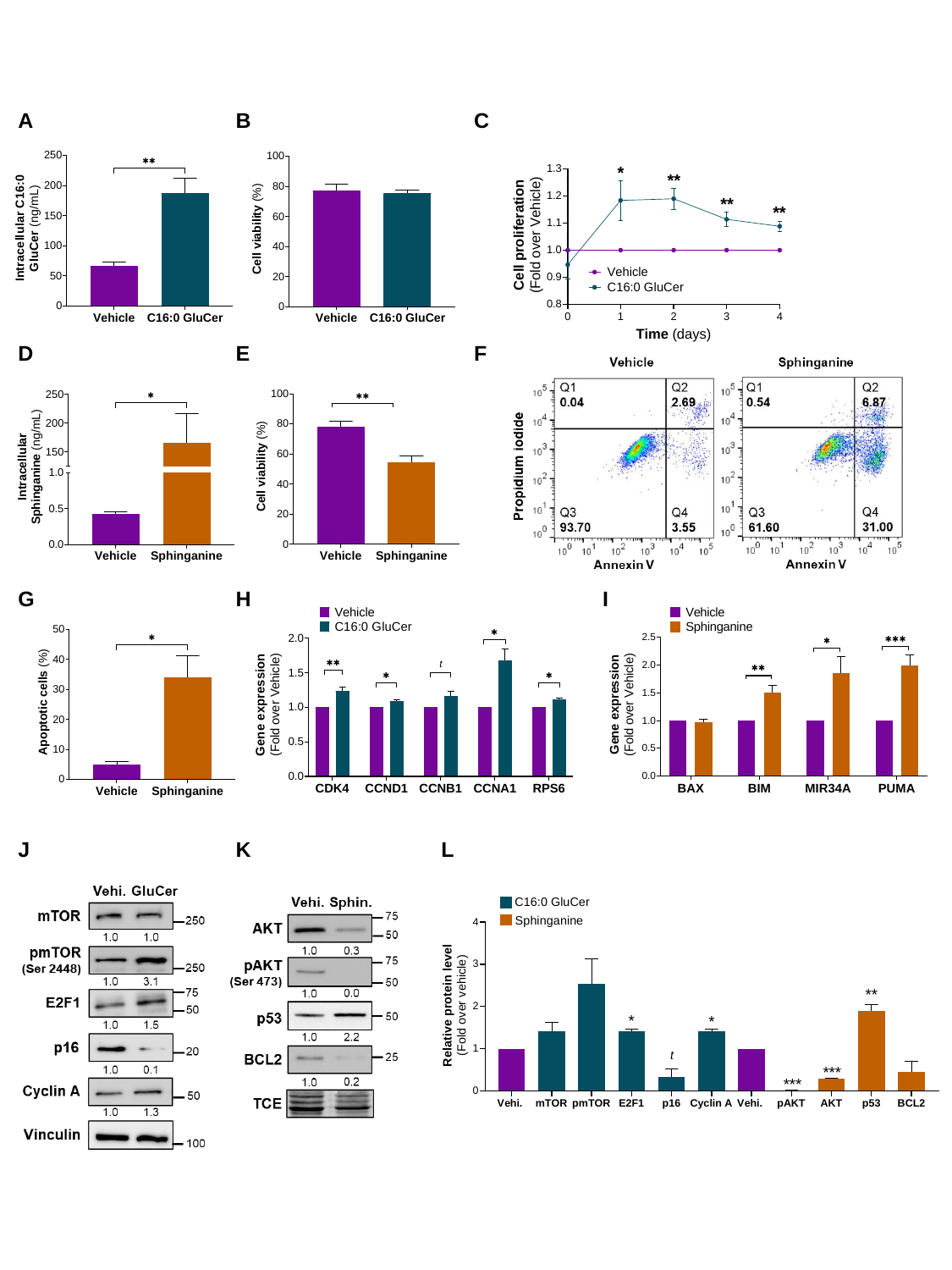

A
B
C
D
E
F
G
H
I
J
K
L

## Slide 7
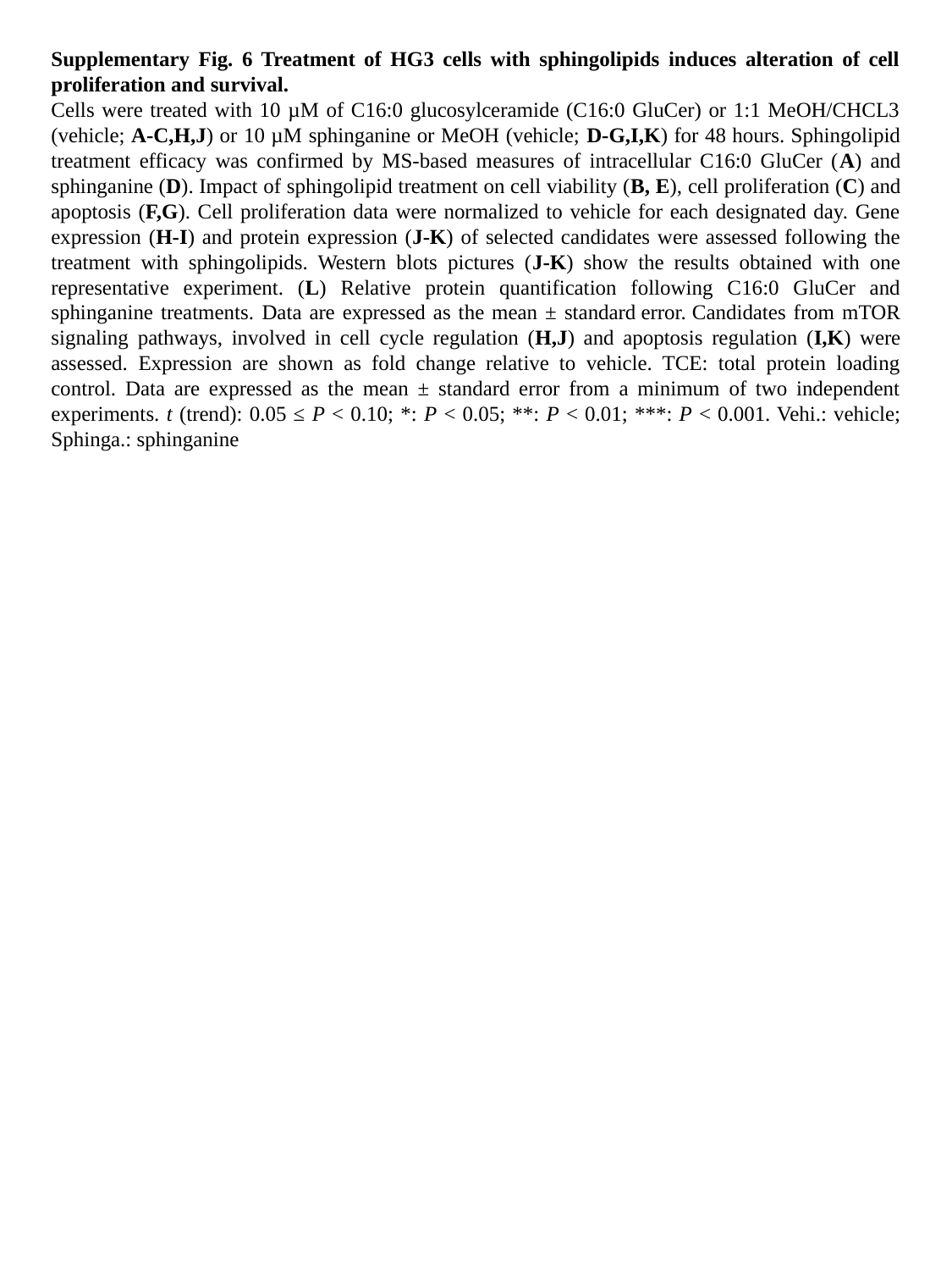

Supplementary Fig. 6 Treatment of HG3 cells with sphingolipids induces alteration of cell proliferation and survival.
Cells were treated with 10 µM of C16:0 glucosylceramide (C16:0 GluCer) or 1:1 MeOH/CHCL3 (vehicle; A-C,H,J) or 10 µM sphinganine or MeOH (vehicle; D-G,I,K) for 48 hours. Sphingolipid treatment efficacy was confirmed by MS-based measures of intracellular C16:0 GluCer (A) and sphinganine (D). Impact of sphingolipid treatment on cell viability (B, E), cell proliferation (C) and apoptosis (F,G). Cell proliferation data were normalized to vehicle for each designated day. Gene expression (H-I) and protein expression (J-K) of selected candidates were assessed following the treatment with sphingolipids. Western blots pictures (J-K) show the results obtained with one representative experiment. (L) Relative protein quantification following C16:0 GluCer and sphinganine treatments. Data are expressed as the mean ± standard error. Candidates from mTOR signaling pathways, involved in cell cycle regulation (H,J) and apoptosis regulation (I,K) were assessed. Expression are shown as fold change relative to vehicle. TCE: total protein loading control. Data are expressed as the mean ± standard error from a minimum of two independent experiments. t (trend): 0.05 ≤ P < 0.10; *: P < 0.05; **: P < 0.01; ***: P < 0.001. Vehi.: vehicle; Sphinga.: sphinganine

## Slide 8
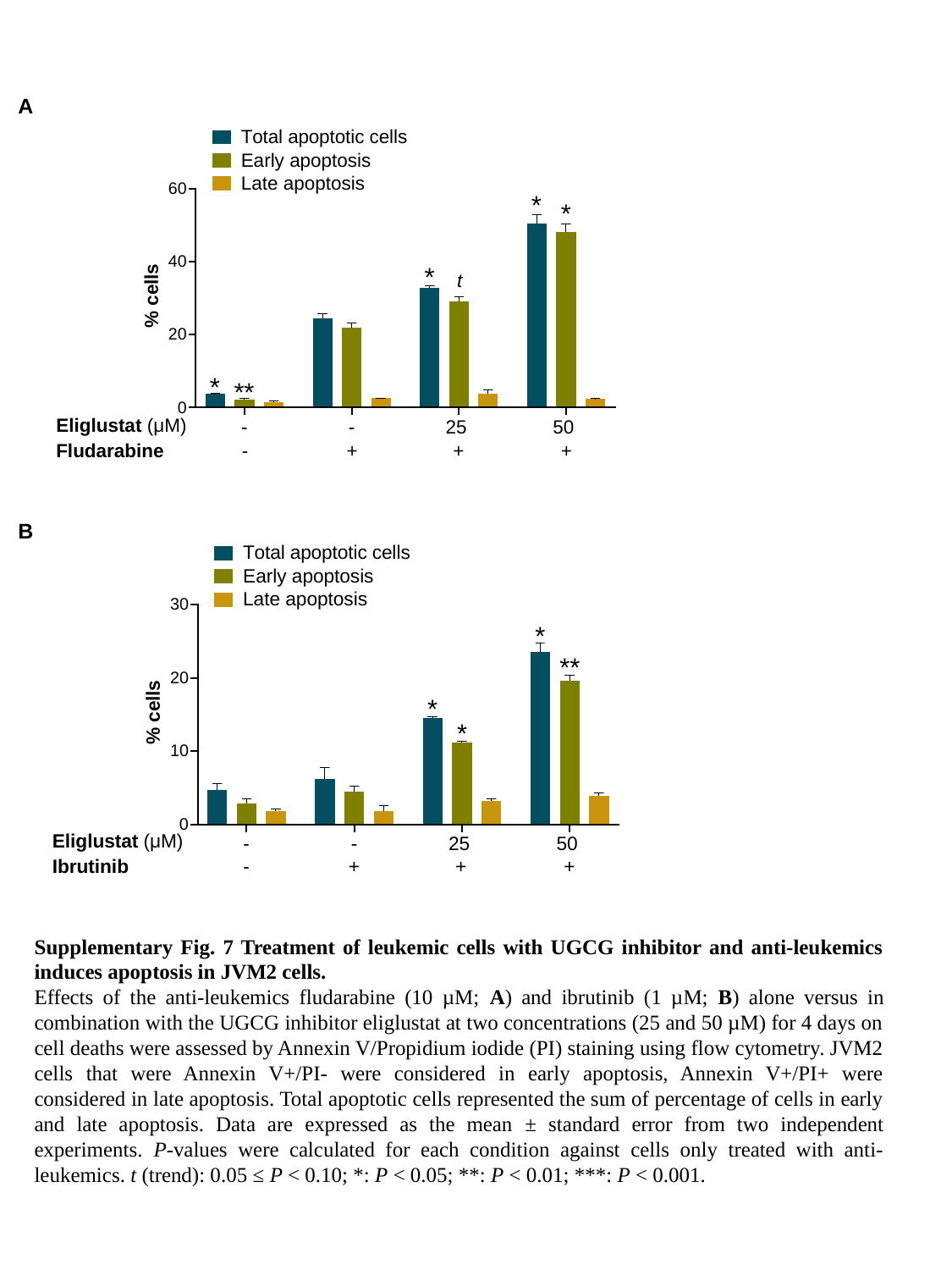

A
B
Supplementary Fig. 7 Treatment of leukemic cells with UGCG inhibitor and anti-leukemics induces apoptosis in JVM2 cells.
Effects of the anti-leukemics fludarabine (10 µM; A) and ibrutinib (1 µM; B) alone versus in combination with the UGCG inhibitor eliglustat at two concentrations (25 and 50 µM) for 4 days on cell deaths were assessed by Annexin V/Propidium iodide (PI) staining using flow cytometry. JVM2 cells that were Annexin V+/PI- were considered in early apoptosis, Annexin V+/PI+ were considered in late apoptosis. Total apoptotic cells represented the sum of percentage of cells in early and late apoptosis. Data are expressed as the mean ± standard error from two independent experiments. P-values were calculated for each condition against cells only treated with anti-leukemics. t (trend): 0.05 ≤ P < 0.10; *: P < 0.05; **: P < 0.01; ***: P < 0.001.
