# Supplementary material for: Untargeted metabolomics identifies metabolic dysregulation of sphingolipids associated with aggressive chronic lymphocytic leukaemia and poor survival
Source: Clin Transl Med. 2023 Nov 30;13(12):e1442. doi: 10.1002/ctm2.1442 (PMC10689972; doi:10.1002/ctm2.1442)
Supplement: Supplementary file 2 — Supporting Information [file CTM2-13-e1442-s002.docx]

**Untargeted metabolomics identifies metabolic dysregulation of sphingolipids associated with aggressive chronic lymphocytic leukemia and poor survival**

Flora Nguyen Van Long^1^, Délya Valcourt-Gendron^1^, Patrick Caron^1^, Michèle Rouleau^1^, Lyne Villeneuve^1^, David Simonyan^2^, Trang Le^3^, Roxanne Sergerie^1^, Isabelle Laverdière^1^, Katrina Vanura^3^ and Chantal Guillemette^1,4^

Affiliations

^1^Centre Hospitalier Universitaire de Québec Research Center - Université Laval (CRCHUQc-UL), Faculty of Pharmacy and Centre de Recherche sur le Cancer (CRC-UL), Université Laval, Québec, Canada

^2^Statistical and Clinical Research Platform, CRCHUQc-UL, Québec, Canada
^3^Department of Medicine I, Division of Haematology and Haemostaseology, Medical University of Vienna, Vienna, Austria.

^4^Canada Research Chair in Pharmacogenomics

Correspondence: [chantal.guillemette@crchudequebec.ulaval.ca](mailto:chantal.guillemette@crchudequebec.ulaval.ca%E2%80%AF)

This file includes:

Supplementary material and methods

Supplementary Tables 1-2, 4-8 and 10.

References for Supplementary material and methods

Supplementary Tables 3 and 9 are provided as separate excel files

**Supplementary material and methods**

**Chemicals**

Methanol (MeOH), isopropyl alcohol (IPA), acetonitrile (ACN), ethyl acetate (EtOAc), chloroform and formic acid (FA) were obtained from VWR Canlab (Montreal, QC, Canada). C16 ceramide d18:1/16:0 (C16:0 Cer), C24:1 Ceramide d18:1/24:1 (C24:1 Cer), C24:1 Glucosyl (β) Ceramide d18:1/24:1 (C24:1 GluCer) and C16 glucosyl (β) Ceramide d18:1/16:0 (C16:0 GluCer) were purchased from Avanti Polar Lipids (Alabaster, AL, USA). d-Erythro-sphingosine (d18:1 sphingosine), d-Erythro-C18 Dihydro d-sphingosine (d18:0 sphinganine) and sphingosine-1-phosphate (d18:1-S1P) were purchased from TRC (North York, ON, Canada). Internal standards d-erythro-sphingosine-d7 (d18:1 sphingosine-d7) and C16 ceramide d18:1-d7/16:0 (C16:0 Cer-d7) were purchased from Avanti Polar Lipids. Eliglustat and ibrutinib were purchased from Selleck Chemicals (Houston, TX, USA). Ibiglustat was obtained from Cayman Chemical (Ann Arbor, MI, USA), ibrutinib and fludarabine from Sigma-Aldrich (Burlington, MA, USA).

**Untargeted metabolomics and lipidomics, and MS assay targeting sphingolipids**

Five biological replicates for each cell line (80 × 10^6^ cells) were profiled on Metabolon’s global metabolomic platform (HD4) using ultra-high-performance liquid chromatography–tandem mass spectroscopy (UPLC-MS/MS). Briefly, to recover the metabolites, samples were prepared using the MicroLab Star system (Hamilton Company, Reno, NV, USA). The extracts were divided into four fractions: three were subjected to reversed-phase UPLC-MS/MS in both the positive ion mode (two fractions) and negative ion mode (one fraction) electrospray ionization and to analysis by hydrophilic interaction ultra-performance liquid chromatography (HILIC)/UPLC-MS/MS with negative ion mode (one fraction). Compound identification was done through comparison of ion features of experimental samples with a reference library of chemical standard entries that included: retention time, molecular weight (*m/z*), preferred adducts, and in-source fragments and associated MS spectra.^1,2^ Peaks were quantified using the area under the curve method, and data were normalized for inter-day signal differences. Enrichment scores for pathway enrichment analysis were calculated using the formula : (k/m)/[(n-k)/(N-m)] in which k refers to the number of significant metabolites altered in the pathway of interest, m refers to the number of total metabolites identified in the pathway of interest, n to the total number of significantly altered metabolites in the dataset and N to the total metabolites identified in the dataset.

Lipids were profiled using Metabolon’s Complex Lipid Panel on five biological replicates for each cell line (80 × 10^6^ cells). Lipids were extracted by a mix of methanol, water and dichloromethane in the presence of internal standards. MS was performed with the Shimadzu LC with nano PEEK tubing and a Sciex Selexlon-5 500 QTRAP. Samples were analyzed in both positive and negative ion mode electrospray. Lipids were quantified by using the ratio of the signal intensity for each metabolite relative to that of the assigned internal standard, multiplied by the concentration of each internal standard added to the samples.

For the targeted analysis of sphingolipids by HPLC coupled to tandem mass spectrometry (LC-MS/MS), we used primary stock solutions for sphingolipid reference standards and internal standards prepared in chloroform/methanol (2:1, v/v) and stored at –20°C. Working standard solutions for calibration curves were prepared by diluting stock solutions in methanol. Seven non-zero standards were prepared on the day of the assay in a mix of charcoal-stripped serum to achieve a specific concentration needed for the calibration: from 125 to 5 000 ng/ml for C24:1 Cer, C16:0 GluCer, C24:1 GluCer and d18:1-S1P; from 75 to 3 000 ng/ml for C16:0 Cer; from 1 to 40 ng/ml for d18:0 sphinganine and from 2.5 to 100 ng/ml for d18:1 sphingosine. The internal standard intermediate solution was prepared in MeOH at 200 ng/ml and was stored at –20°C. Standard curve samples (prepared in charcoal-stripped serum) and CLL samples (25 µl) were individually placed in microcentrifuge tubes, and 1 ml of IPA/water (80:20, v/v) was added to each tube. Working solutions (25 µl) were added to the standard curve samples. Each sample was mixed with 25 µl of the internal standard intermediate solution or with 25 µl water for the blank. Samples were centrifuged at 14 000 rpm for 10 min. For each sample, the supernatant was transferred to a glass tube and evaporated at 35°C to dryness under nitrogen with a Turbo Vap (Zymark Corporation, Hopkinton, MA, USA). The residue was reconstituted with 200 µl MeOH and transferred to a spin filter (Canadian Life Science, Peterborough, ON, Canada) with 0.2-μm mesh through which it was filtered at 3 000 rpm for 5 min. The eluate was transferred to a 96-well plate prior to analysis. The chromatographic separation of ceramides was achieved using HPLC at ambient temperature on a Nexera system with a run duration of 20 min (Shimadzu Scientific Instrument Inc., Columbia, MD, USA). Separation was performed on an Ascentis Express C18 column (75 × 2.1 mm ID; particle size, 2.7 μm; Sigma, Oakville, ON, Canada). A binary mobile phase, consisting of water containing 0.1% FA (solvent A) and MeOH/ACN/IPA (4:1:1, v/v) containing 0.1% FA (solvent B), was used at a flow rate of 0.3 ml/min. The analytes were eluted using the following program: 0–4.0 min, linear gradient 60–100% B; 4.0–15 min, isocratic 100% B; 15.0–15.1 min, linear gradient 100–60% B; 15.1–20 min, isocratic 60% B to re-equilibrate the column. The samples were quantified on a 6 500 LC-MS/MS system (AB Sciex, Concord, ON, Canada) equipped with a turbo ion-spray source, configured in multiple reaction monitoring mode. Electrospray ionization was performed in the positive mode with a probe temperature of 350°C. The nebulization and the collision gas were set at 30 and medium, respectively. Analyst software version 1.7.2 (AB Sciex) was used for system control and data analysis. Transitions and energies are listed in **Supplementary Table 8**. Quantitative metabolomics data generated and analyzed for untargeted, targeted and lipodomics approaches in leukemic B-cells and human samples are provided in **Supplementary Table 9**.

**Cell models and culture**

All cell culture components were purchased from Wisent Bioproducts (St-Bruno, QC, Canada). Cell lines were regularly tested for mycoplasma contamination, with the most recent test performed on March 12^th^ 2023. The NCBI BioSample database (https://www.ncbi.nlm.nih.gov/biosample) was used to verify that all leukemic cell lines used in this study were not misidentified or contaminated by other human cell lines. Cell lines (MEC1 and JVM2) engineered to overexpress UGT2B17, described previously,^3^ were maintained in RPMI medium supplemented with 10% fetal bovine serum (FBS) for JVM2 and MEC1 or with 15% FBS for HG3, 1% penicillin/streptomycin, 1% sodium pyruvate and 1% l-glutamine in 5% CO_2_ at 37°C, as recommended by the supplier DSMZ (Braunschweig, Germany). The growth medium contained 10 μg/ml blasticidin (MEC1-UGT2B17^OE^) or 4 μg/ml puromycin (MEC1 controls) and 5 μg/mL blasticidin for JVM2 cells. Primary CLL cells were cultured in RPMI 1640 GlutaMAX medium supplemented with 25 mM HEPES, 1 mM sodium pyruvate and 10% heat-inactivated FBS. All cell culture components for CLL patient cells were purchased from ThermoFisher Scientific (Waltham, MA, USA).

The HG3-UGT2B17 knockout line (HG3-UGT2B17^KO^) was generated by the CRISPR/Cas9 gene mutagenesis. The sgRNA targeting exon 1 of *UGT2B17* and designed using the Broad Institute Gene Perturbation Platform sgRNA Designer and the CRISPR targets of the UCSC browser (accessed in July 2020), displayed the highest efficiency for *UGT2B17*, had the lowest number of off-target genes and did not target the highly similar gene *UGT2B15*. sgRNA oligonucleotides were synthesized by IDT (Integrated DNA Technology, Coralville, IA, USA) and then were cloned into pLKO5.sgRNA.EFS.GFP, a gift from Benjamin Ebert (Addgene plasmid #57822), as described.^4^ Lentiviral production was carried out in LentiX 293T cells (Takara Bio, San José, CA, USA) maintained in DMEM supplemented with 10% FBS, 1% sodium pyruvate and 1% l-glutamine. Cells were co-transfected with pLKO5.sgRNA.EFS.GFP, pPAX2 (Addgene #12260) and pMD2.G (Addgene #12259) using Lipofectamine 2 000 (Invitrogen, Eugene, OR, USA) as per the manufacturer’s instructions. Viral supernatants were collected at 48 h and 72 h after transfection. The pooled supernatants were filtered through a 0.45-μm filter to remove debris, concentrated overnight with 4% polyethylene glycol 10 000 (Sigma-Aldrich, Burlington, MA, USA) prepared in phosphate-buffered saline and recovered by centrifugation at 1 500 ×*g* for 30 min. Lentiviruses were transduced for 72 h in HG3-Cas9 cells, which constitutively express Cas9, a kind gift of Jesús María Hernández-Rivas (University of Salamanca, Cancer Research Center, Salamanca, Spain).^5^ Clones of individual GFP-expressing cells were generated by FACS sorting followed by amplification in culture. Genomic DNA was extracted using a QiaAmp DNA blood mini kit (Qiagen**,** Toronto, ON, Canada) and screened by TIDE (<https://tide.nki.nl/>)^6^ to identify open reading frame–disrupting insertions or deletions produced by the sgRNA. The sequences for the sgRNA that successfully produced UGT2B17 KO cells, for the control non-targeting sgRNA^5^ and for the TIDE primers are provided in **Supplementary Table 10**. Clones were subsequently generated by FACS-sorted single-cell distribution in 96-well plates; they were then amplified and Sanger sequenced to characterize insertions or deletions in the region of interest. Cells were maintained with medium containing 5 μg/ml blasticidin. The absence of UGT2B17 expression was confirmed using a functional assay consisting of measuring glucuronide formation with dihydrotestosterone as a substrate.^3^

**Cell-based assays**

For proliferation assays, JVM2 and HG3 cells were plated at a density of 1 × 10^4^ cells/well in 96-well U-bottom tissue culture plates (BD Bioscience, Franklin Lakes, NJ, USA). For treatment assays with sphingolipids, growth medium was supplemented with sphinganine or C16:0 GluCer or the appropriate vehicle for 48 h before plating. Every 24 h, 20 μl of CellTiter aqueous one solution cell proliferation reagent (MTS Promega, Madison, WI, USA) was added to each well. For drug treatment assays, growth medium was supplemented with UGCGi or vehicle at the time of plating and renewed every 48 h. After 96 h of treatment, 20 μl of CellTiter was added. For co-treatment assays with drugs, UGCGi and anti-leukemics or the appropriate vehicle were included in the growth medium at the time of plating; then, after 96 h, 20 μl of CellTiter was added. Absorbance at 490 nm was measured in the Infinite M1000 plate reader (Tecan, Männedorf, Switzerland) after a 4-h incubation with CellTiter in the cell culture incubator at 37°C. Assays were replicated at least three times, in triplicate. Cell viability after 48 h of treatment with sphingolipids was also measured by staining an aliquot of cells with 50% trypan blue and measuring the number of stained cells with a TC-10 automated cell counter (Bio-Rad, Hercules, CA, USA). Apoptosis assays were carried out on cells treated with 10 μM C16:0 GluCer or sphinganine or the appropriate vehicle for 48 h. Aliquots of 1 × 10^6^ cells were washed with Dulbecco’s Phosphate Buffered Saline (Wisent Bioproducts) once before being resuspended in Annexin V binding buffer (Invitrogen). Cells were then stained with Pacific blue Annexin V according to the manufacturer’s instructions (Invitrogen) and propidium iodide (4 ng/ml, Sigma-Aldrich) in the dark at room temperature for 12 min. Data acquisition of flow cytometry experiments was performed on the BD FACSCelesta^TM^ flow cytometer (BD Biosciences, Ashland, OR, USA) and using BD FACSDivaTM software version 8.0.1.1 (Becton, Dickinson and Company, 2019). Data analysis was carried out using FlowJo v10.8.1 software (BD Biosciences).

CLL patient cells were plated at a density of 3.3 × 10^6^ cells/mL in 12-well plates and treated with 10 μM C16:0 GluCer or vehicle for 22 h and with 10 μM sphinganine or vehicle for 4 h. Cell viability was assessed using the CellTiter-Blue Cell Viability Assay (Promega) and apoptosis was determined using DAPI (4',6-diamidino-2-phenylindole, Sigma-Aldrich) and APC Annexin V (BioLegend, San Diego, CA, USA) staining as described previously.^7^ Data acquisition and analysis of flow cytometry experiments were performed on a Beckman Coulter Cytoflex (Beckman Coulter, Brea, CA, USA) flow cytometer and using CytExpert software (Beckman Coulter).

**Gene and protein expression analyses**

Total RNA was extracted from cells treated with 10 μM C16:0 GluCer or vehicle for 48 h using RNeasy Plus Mini kits and the QIAcube system (Qiagen). Reverse transcription was carried out using SuperScript IV reverse polymerase (Thermo Fisher Scientific, Waltham, MA, USA) on the GeneAmp PCR System 9700 Thermal Cycler (Thermo Fischer). Gene expression was measured by qPCR on the StepOnePlus Real Time PCR system (Thermo Fischer) with 10 ng cDNA using the Power SYBR Green PCR Master Mix reagent (Thermo Fischer). Data acquisition was performed using the StepOne Software v2.3 (Thermo Fischer). Relative expression levels were calculated using the 2^–∆∆CT^ method and normalized with *36B4* as the reference gene. Primer sequences are provided in **Supplementary Table 10**.

For analysis of protein expression, cells were collected, washed and then lysed in lysis buffer consisting of 150 mM NaCl, 50 mM Tris-HCl (pH 7.4), 0.3% sodium deoxycholate, 1% IGEPAL CA-630 (Sigma- Aldrich), 1 mM EDTA, protease and phosphatase inhibitors (Sigma-Aldrich). Protein concentration was quantified using the Pierce BCA Protein Assay Kit (Thermo Fisher Scientific, Rockford, IL, USA). Protein samples (50 μg), were mixed with Laemmli buffer (Bio-Rad, Mississauga, ON, CA), heated at 95°C for 5 min, separated on a 7.5% or 12% SDS-polyacrylamide gel and transferred to nitrocellulose membranes using the Trans-Blot Turbo Transfer system (Bio-Rad). Membranes were blocked in phosphate-buffered saline containing 0.2% IGEPAL and 5% dry milk for 1 h at room temperature and then were incubated with antibodies overnight at 4°C. Antibodies directed against AKT (#4691), pAKT (Ser473, #4060), mTOR (#2983), pmTOR (Ser2448, #5536) and FOXO1 (#2880T) were diluted at 1:1 000 and purchased from Cell Signaling Technology (Belleville, ON, Canada). Anti-p53 (sc-126) and anti-BCL2 from Santa Cruz Biotechnology (Dallas, TX, USA) were diluted at 1:500 and anti-BAX (#50599-2-Ig, 1:2 000) was purchased from Proteintech (Rosemont, IL, USA). As the loading control, antibodies against GAPDH (#90945, 1:80 000), vinculin (V9131, 1:10 000) from Sigma Aldrich as well as total protein stain (2,2,2-trichloro-ethanol; Sigma-Aldrich, 1:200) were used. Proteins were detected using horseradish peroxidase–linked anti–rabbit IgG from donkey (1:10 000; #45000682; Thermo Fisher) and a horseradish peroxidase anti-mouse IgG from goat (1:5000; #115-035-146; Jackson Immunoresearch, West Grove, PA, USA) and Clarity or Clarity Max Western ECL Substrate (Bio-Rad). The chemiluminescence signal was detected with the ChemiDoc Imaging system (Bio-Rad) and analyzed using Image Lab software (v 6.0.1, Bio-Rad). For western blot images, one representative loading control was presented. Normalization of each protein expression was performed using each loading control that was on the same membrane as the protein quantified.

**Supplementary Tables 1-2, 4-8 and 10**

**Supplementary Table 1.** Characteristics of CLL patients studied to establish the relationships between circulating sphingolipid levels and treatment-free survival (TFS), and the biological impact of sphingolipids.

|  | **Plasma samples**  **(N=107)** | | **CLL cells**  **(N=3)** | |
| --- | --- | --- | --- | --- |
|  | **N** | **(%)** | **N** | **(%)** |
| **Age**  (median, min-max years) | 62.4 (25.3 – 83.6) | | 57.8 (47.2 – 62.8) | |
| **Sex** | | | | |
| Female | 44 | (41.1) | 1 | (33.3) |
| Male | 63 | (58.9) | 2 | (66.7) |
| **IGHV mutational status** | | | | |
| N/A | 16 | (15.0) | 1 | (33.3) |
| Mutated | 48 | (44.8) | 2 | (66.7) |
| Unmutated | 43 | (40.2) |  |  |
| **Binet Stage** | | | | |
| N/A | 3 | (2.8) |  |  |
| A | 92 | (86.0) | 3 | (100.0) |
| B or C | 12 | (11.2) |  |  |
| **UGT2B17 expression** | | | | |
| N/A | 1 | (0.9) | 1 | (33.3) |
| Low | 66 | (61.7) | 2 | (66.7) |
| High | 40 | (37.4) |  |  |
| **CD38 expression** |  |  |  |  |
| N/A | 7 | (6.5) |  |  |
| Low | 70 | (65.4) | 1 | (33.3) |
| High | 30 | (28.0) | 2 | (66.7) |
| **Del11q** |  |  |  |  |
| N/A | 5 | (4.7) |  |  |
| Negative | 83 | (77.6) | 1 | (33.3) |
| Positive | 19 | (17.8) | 2 | (66.7) |
| **Trisomy 12** |  |  |  |  |
| N/A | 5 | (4.7) |  |  |
| Negative | 91 | (85.0) | 3 | (100.0) |
| Positive | 11 | (10.3) |  |  |
| **Del13q** |  |  |  |  |
| N/A | 5 | (4.7) |  |  |
| Negative | 50 | (46.7) | 1 | (33.3) |
| Positive | 52 | (48.6) | 2 | (66.7) |
| **Del17p** |  |  |  |  |
| N/A | 5 | (4.7) |  |  |
| Negative | 95 | (88.8) | 3 | (100.0) |
| Positive | 7 | (6.5) |  |  |

N/A: data not available; Del11q: 11q chromosome deletion; Del13q: 13q chromosome deletion; Del17p : 17p chromosome deletion.

**Supplementary Table 2.** Top 10 most altered metabolic pathways associated with UGT2B17 overexpression (OE) in leukemic B-cell lines.

| **Subpathway** | **Superpathway** | **Enrichment score** |
| --- | --- | --- |
| **MEC1-UGT2B17^OE^ vs. control** |  |  |
| Purine and Pyrimidine Metabolism | Nucleotide | 5.1 |
| Drug - Antibiotic | Xenobiotics | 5.1 |
| Pentose Phosphate Pathway | Carbohydrate | 5.1 |
| Folate Metabolism | Co-factors and Vitamins | 5.1 |
| Glycerolipid Metabolism | Lipid | 3.4 |
| Gamma-Glutamyl Amino Acid | Carbohydrate | 3.2 |
| Pentose Metabolism | Nucleotide | 3.0 |
| Purine Metabolism, (Hypo)Xanthine/Inosine containing | Nucleotide | 2.9 |
| Fructose, Mannose and Galactose Metabolism | Carbohydrate | 2.6 |
| Creatinine Metabolism | Amino Acid | 2.6 |
| **JVM2-UGT2B17^OE^ vs. control** |  |  |
| Phosphatidylserine | Lipid | 2.1 |
| Fatty Acid Metabolism (Acyl Choline) | Lipid | 2.1 |
| Disaccharides and Oligosaccharides | Carbohydrate | 2.1 |
| Purine and Pyrimidine Metabolism | Nucleotide | 2.1 |
| Fatty Acid Metabolism (Acyl Carnitine, Short Chain) | Lipid | 2.1 |
| Hemoglobin and Porphyrin Metabolism | Cofactors and Vitamins | 2.1 |
| Tocopherol Metabolism | Cofactors and Vitamins | 2.1 |
| Fatty Acid Synthesis | Lipid | 2.1 |
| Ceramide PE | Lipid | 2.1 |
| Biotin Metabolism | Cofactors and Vitamins | 2.1 |

PE: Phosphoethanolamine

**Supplementary Table 4.** The expression level of genes that encode several sphingolipid biosynthetic pathways was associated with overall survival in the ICGC cohort.

|  | **Overall survival of CLL patients** | | | |
| --- | --- | --- | --- | --- |
| **Gene** | HR | 95%CI | *P*-value | |
| *SGPL1* | 1.3 | 0.7 – 2.3 | 0.43 | |
| *S1PR1* | 1.4 | 0.8 – 2.6 | 0.25 | |
| *S1PR2* | 4.8 | 2.6 – 8.7 | **<0.0001** | |
| *S1PR3* | 1.4 | 0.7 – 2.7 | 0.35 | |
| *S1PR4* | 1.2 | 0.6 – 2.1 | 0.62 | |
| *S1PR5* | 1.1 | 0.6 – 2.0 | 0.82 | |
| *SGPP1* | 1.1 | 0.6 – 2.1 | 0.68 | |
| *SGPP2* | 0.2 | 0.1 – 0.4 | **<0.0001** | |
| *SPHK1* | 1.0 | 0.5 – 1.8 | 0.97 | |
| *SPHK2* | 1.4 | 0.8 – 2.6 | 0.23 | |
| *CERS1* | N/A | | |  |
| *CERS2* | 1.9 | 1.1 – 3.5 | **0.03** | |
| *CERS3* | 0.7 | 0.4 – 1.2 | 0.17 | |
| *CERS4* | 2.0 | 1.1 – 3.6 | **0.02** | |
| *CERS5* | 1.5 | 0.8 – 2.7 | 0.22 | |
| *CERS6* | 2.4 | 1.3 – 4.3 | **0.008** | |
| *ACER1* | 1.1 | 0.6 – 1.9 | 0.84 | |
| *ACER2* | 1.1 | 0.6 – 2.0 | 0.70 | |
| *ACER3* | 1.6 | 0.9 – 3.0 | 0.12 | |
| *CERK* | 1.4 | 0.8 – 2.5 | 0.32 | |
| *UGT8* | 2.7 | 1.5 – 4.9 | **0.002** | |
| *GALC* | 1.5 | 0.8 – 2.7 | 0.18 | |
| *UGCG* | 2.0 | 1.1 – 3.7 | **0.02** | |
| *GBA* | 0.9 | 0.5 – 1.6 | 0.70 | |
| *GBA2* | 1.0 | 0.5 – 1.8 | 0.97 | |
| *GLB1* | 0.6 | 0.3 – 1.1 | 0.14 | |
| *B4GALT6* | 1.0 | 0.5 – 2.1 | 0.92 | |
| *SGMS1* | 1.3 | 0.7 – 2.4 | 0.35 | |
| *SGMS2* | 2.5 | 1.4 – 4.5 | **0.006** | |
| *SMPD1* | 1.2 | 0.7 – 2.2 | 0.54 | |
| *SMPD2* | 1.6 | 0.9 – 2.9 | 0.12 | |
| *SMPD3* | 0.6 | 0.3 – 1.1 | *0.09* | |
| *SPTSSA* | 4.4 | 2.4 – 8.0 | **<0.0001** | |
| *SPTLC1* | 1.2 | 0.7 – 2.2 | 0.55 | |
| *SPTLC2* | 1.2 | 0.7 – 2.3 | 0.48 | |
| *SPTLC3* | 1.1 | 0.6 – 2.2 | 0.79 | |
| *KDSR* | 1.5 | 0.8 – 2.8 | 0.18 | |
| *DEGS1* | 1.1 | 0.6 – 2.1 | 0.68 | |
| *DEGS2* | 2.4 | 1.3 – 4.4 | **0.005** | |
| *ST3GAL5* | 1.4 | 0.7 – 2.6 | 0.35 | |
| *ST8SIA1* | 1.5 | 0.8 – 3.0 | 0.20 | |
| *B4GALNT1* | N/A | | |  |
| *B3GALT4* | 1.1 | 0.6 – 2.0 | 0.71 | |
| *ST3GAL1* | 0.9 | 0.5 – 1.7 | 0.80 | |
| *A4GALT* | 0.8 | 0.4 – 1.7 | 0.62 | |
| *ST3GAL2* | 1.5 | 0.8 – 2.7 | 0.19 | |
| *FUT1* | 1.4 | 0.6 – 2.9 | 0.38 | |
| *FUT2* | 1.0 | 0.5 – 1.8 | 0.89 | |
| *FUT3* | 1.8 | 1.0 – 3.3 | *0.055* | |
| *FUT4* | 2.0 | 1.1 – 3.7 | **0.030** | |
| *FUT5* | 1.4 | 0.8 – 2.6 | 0.25 | |
| *FUT6* | 1.1 | 0.6 – 1.9 | 0.86 | |
| *FUT7* | 1.4 | 0.8 – 2.6 | 0.26 | |
| *FUT8* | 0.7 | 0.4 – 1.3 | 0.32 | |
| *FUT9* | 0.8 | 0.4 – 1.5 | 0.44 | |
| *FUT10* | 0.9 | 0.5 – 1.6 | 0.61 | |
| *FUT11* | 1.0 | 0.6 – 1.8 | 0.99 | |
| *B3GNT5* | 1.3 | 0.7 – 2.4 | 0.35 | |
| *B3GALT1* | 0.8 | 0.4 – 1.5 | 0.44 | |

HR: hazard ratio; 95%CI: 95% confidence interval; N/A: data not available. Significant results are highlighted in bold and *P*-values defined as trend: 0.05 ≤ *P* < 0.10 are italicized.

**Supplementary Table 5.** The expression level of genes that encode several sphingolipid biosynthetic pathways was associated with overall survival in women of the ICGC cohort.

| **Overall survival of female CLL patients** | | | |
| --- | --- | --- | --- |
| **Gene** | HR | 95%CI | *P*-value |
| *SGPL1* | 0.4 | 0.1 – 1.3 | 0.14 |
| *S1PR1* | 0.7 | 0.2 – 2.0 | 0.48 |
| *S1PR2* | 11.3 | 3.8 – 33.6 | **0.003** |
| *S1PR3* | 1.7 | 0.5 – 5.7 | 0.36 |
| *S1PR4* | 1.4 | 0.5 – 4.0 | 0.58 |
| *S1PR5* | 0.3 | 0.1 – 0.8 | **0.014** |
| *SGPP1* | 1.5 | 0.5 – 4.5 | 0.45 |
| *SGPP2* | 0.3 | 0.1 – 0.8 | **0.022** |
| *SPHK1* | 0.6 | 0.2 – 1.7 | 0.25 |
| *SPHK2* | 1.7 | 0.6 – 5.0 | 0.36 |
| *CERS1* | N/A | | |
| *CERS2* | 0.6 | 0.2 – 1.8 | 0.38 |
| *CERS3* | 1.6 | 0.5 – 4.7 | 0.41 |
| *CERS4* | 3.6 | 1.2 – 10.9 | **0.022** |
| *CERS5* | 0.9 | 0.3 – 2.6 | 0.78 |
| *CERS6* | 6.3 | 2.1 – 18.7 | **0.006** |
| *ACER1* | 1.7 | 0.6 – 5.0 | 0.36 |
| *ACER2* | 0.9 | 0.3 – 2.7 | 0.88 |
| *ACER3* | 2.0 | 0.7 – 5.9 | 0.24 |
| *CERK* | 1.4 | 0.5 – 4.2 | 0.53 |
| *UGT8* | 2.8 | 0.9 – 8.3 | *0.073* |
| *GALC* | 0.6 | 0.2 – 1.9 | 0.42 |
| *UGCG* | 2.6 | 0.9 – 7.8 | *0.101* |
| *GBA* | 0.8 | 0.3 – 2.3 | 0.64 |
| *GBA2* | 0.6 | 0.2 – 1.8 | 0.35 |
| *GLB1* | 0.4 | 0.1 – 1.2 | 0.13 |
| *B4GALT6* | 0.9 | 0.3 – 2.7 | 0.78 |
| *SGMS1* | 1.8 | 0.6 – 5.5 | 0.30 |
| *SGMS2* | 1.5 | 0.5 – 4.6 | 0.45 |
| *SMPD1* | 0.6 | 0.2 – 1.9 | 0.38 |
| *SMPD2* | 1.1 | 0.4 – 3.2 | 0.92 |
| *SMPD3* | 0.9 | 0.3 – 2.5 | 0.78 |
| *SPTSSA* | 2.6 | 0.9 – 7.6 | *0.098* |
| *SPTLC1* | 0.8 | 0.3 – 2.3 | 0.65 |
| *SPTLC2* | 1.3 | 0.5 – 4.0 | 0.60 |
| *SPTLC3* | 0.9 | 0.3 – 3.0 | 0.91 |
| *KDSR* | 0.3 | 0.1 – 0.8 | **0.029** |
| *DEGS1* | 0.8 | 0.3 – 2.5 | 0.74 |
| *DEGS2* | 2.0 | 0.6 – 6.0 | 0.20 |
| *ST3GAL5* | 0.5 | 0.2 – 1.4 | 0.21 |
| *ST8SIA1* | N/A | | |
| *B4GALNT1* | N/A | | |
| *B3GALT4* | 1.1 | 0.4 – 3.2 | 0.88 |
| *ST3GAL1* | 0.8 | 0.3 – 2.4 | 0.68 |
| *A4GALT* | 1.4 | 0.3 – 5.7 | 0.62 |
| *ST3GAL2* | 1.1 | 0.4 – 3.3 | 0.86 |
| *FUT1* | 1.9 | 0.4 – 9.0 | 0.33 |
| *FUT2* | 1.9 | 0.6 – 5.6 | 0.28 |
| *FUT3* | 2.5 | 0.8 – 7.4 | 0.10 |
| *FUT4* | 1.1 | 0.4 – 3.1 | 0.94 |
| *FUT5* | 1.3 | 0.4 – 3.8 | 0.65 |
| *FUT6* | 0.7 | 0.3 – 2.2 | 0.57 |
| *FUT7* | 1.3 | 0.4 – 3.8 | 0.66 |
| *FUT8* | 1.3 | 0.4 – 3.9 | 0.63 |
| *FUT9* | N/A | | |
| *FUT10* | 0.6 | 0.2 – 1.9 | 0.41 |
| *FUT11* | 0.7 | 0.2 – 2.1 | 0.53 |
| *B3GNT5* | 0.5 | 0.2 – 1.4 | 0.19 |
| *B3GALT1* | 0.9 | 0.3 – 2.9 | 0.88 |

HR: hazard ratio; 95%CI: 95% confidence interval; N/A: data not available. Significant results are highlighted in bold and *P*-values defined as trend: 0.05 ≤ *P* < 0.10 are italicized.

**Supplementary Table 6.** The expression level of genes that encode several sphingolipid biosynthetic pathways was associated with overall survival in men of the ICGC cohort.

|  | **Overall survival of male CLL patients** | | |
| --- | --- | --- | --- |
| Gene | HR | 95%CI | *P*-value |
| *SGPL1* | 1.8 | 0.9 – 3.7 | 0.13 |
| *S1PR1* | 1.8 | 0.9 – 3.6 | 0.13 |
| *S1PR2* | 3.8 | 1.9 – 7.8 | **0.0007** |
| *S1PR3* | 1.3 | 0.6 – 2.9 | 0.57 |
| *S1PR4* | 1.1 | 0.5 – 2.2 | 0.85 |
| *S1PR5* | 1.4 | 0.7 – 2.6 | 0.35 |
| *SGPP1* | 1.2 | 0.6 – 2.5 | 0.61 |
| *SGPP2* | 0.2 | 0.1 – 0.5 | **0.0003** |
| *SPHK1* | 1.6 | 0.8 – 3.2 | 0.22 |
| *SPHK2* | 1.3 | 0.7 – 2.7 | 0.43 |
| *CERS1* | N/A | | |
| *CERS2* | 3.1 | 1.5 – 6.2 | **0.004** |
| *CERS3* | 0.5 | 0.2 – 0.9 | **0.037** |
| *CERS4* | 1.8 | 0.9 – 3.7 | *0.102* |
| *CERS5* | 2.8 | 1.4 – 5.7 | **0.014** |
| *CERS6* | 2.3 | 1.1 – 4.8 | **0.029** |
| *ACER1* | 0.9 | 0.5 – 1.9 | 0.80 |
| *ACER2* | 1.2 | 0.6 – 2.4 | 0.67 |
| *ACER3* | 1.9 | 0.9 – 3.9 | *0.085* |
| *CERK* | 1.3 | 0.7 – 2.7 | 0.44 |
| *UGT8* | 2.9 | 1.4 – 6.0 | **0.005** |
| *GALC* | 1.7 | 0.8 – 3.5 | 0.15 |
| *UGCG* | 1.8 | 0.9 – 3.7 | *0.106* |
| *GBA* | 1.0 | 0.5 – 2.1 | 0.95 |
| *GBA2* | 1.3 | 0.6 – 2.6 | 0.52 |
| *GLB1* | 0.7 | 0.3 – 1.4 | 0.35 |
| *B4GALT6* | 0.9 | 0.5 – 1.9 | 0.82 |
| *SGMS1* | 1.0 | 0.5 – 2.0 | 0.97 |
| *SGMS2* | 3.1 | 1.5 – 6.3 | **0.006** |
| *SMPD1* | 1.6 | 0.8 – 3.2 | 0.24 |
| *SMPD2* | 1.9 | 0.9 – 3.8 | *0.095* |
| *SMPD3* | 0.6 | 0.3 – 1.1 | *0.107* |
| *SPTSSA* | 5.7 | 2.8 – 11.6 | **<0.0001** |
| *SPTLC1* | 1.5 | 0.8 – 3.1 | 0.25 |
| *SPTLC2* | 1.2 | 0.6 – 2.5 | 0.58 |
| *SPTLC3* | 1.1 | 0.5 – 2.5 | 0.88 |
| *KDSR* | 2.6 | 1.3 – 5.3 | **0.016** |
| *DEGS1* | 1.4 | 0.7 – 2.8 | 0.41 |
| *DEGS2* | 2.9 | 1.4 – 5.9 | **0.007** |
| *ST3GAL5* | 2.3 | 1.2 – 4.8 | **0.028** |
| *ST8SIA1* | 2.5 | 1.1 – 5.6 | **0.010** |
| *B4GALNT1* | N/A | | |
| *B3GALT4* | 1.4 | 0.7 – 2.8 | 0.42 |
| *ST3GAL1* | 0.9 | 0.4 – 1.8 | 0.77 |
| *A4GALT* | 0.7 | 0.3 – 1.6 | 0.41 |
| *ST3GAL2* | 1.7 | 0.8 – 3.5 | 0.15 |
| *FUT1* | 1.4 | 0.6 – 3.2 | 0.47 |
| *FUT2* | 0.7 | 0.3 – 1.4 | 0.32 |
| *FUT3* | 1.2 | 0.6 – 2.4 | 0.69 |
| *FUT4* | 2.9 | 1.4 – 5.8 | **0.011** |
| *FUT5* | 1.5 | 0.7 – 3.1 | 0.26 |
| *FUT6* | 1.3 | 0.6 – 2.7 | 0.48 |
| *FUT7* | 1.5 | 0.7 – 3.1 | 0.28 |
| *FUT8* | 0.5 | 0.3 – 1.0 | *0.084* |
| *FUT9* | 0.3 | 0.3 – 1.6 | 0.47 |
| *FUT10* | 0.8 | 0.4 – 1.7 | 0.57 |
| *FUT11* | 1.1 | 0.6 – 2.3 | 0.74 |
| *B3GNT5* | 2.5 | 1.2 – 5.2 | **0.015** |
| *B3GALT1* | N/A | | |

HR: hazard ratio; 95%CI: 95% confidence interval; N/A: data not available. Significant results are highlighted in bold and *P*-values defined as trend: 0.05 ≤ *P* < 0.10 are italicized.

**Supplementary Table 7.** Overview of this study and previous studies supporting changes in sphingolipid levels observed in CLL patients.

| **Study cohort** | | **Method** | **Main observations** | **Reference** |
| --- | --- | --- | --- | --- |
| **Blood samples**  -CLL (n=107)      M-CLL (n=49)      UM-CLL (n=43)  -Healthy (n=70) | | Targeted MS | Elevated in CLL vs. healthy:   - C16:0 and C24:1 Cer - C16:0 and C24:1 GluCer   Elevated in cases with adverse prognostic features such as IGHV mutational status:   - C16:0 and C24:1 GluCer | This study |
| **Leukemic cells**  JVM2, MEC1, HG3 | | Untargeted MS  Targeted MS | Elevated sphingolipids associated with adverse marker  Elevated C16:0 GluCer and C24:1 GluCer associated with high UGT2B17 expression |  |
| **PBMC**  CLL (n=8) | | Targeted MS | C24:1 and C16:0 Cer, GluCer, SM predominant in CLL  Activation of BCR pathway by anti-IgM:   - Reduced C16:0 and C24:1 Cer - Elevated C16:0 and C24:1 GluCer | Schwamb *et al.*, 2012 |
| **Blood samples**  -Indolent CLL (n=51)  -Aggressive CLL (n=42) (or requiring treatment)  -Healthy (n=45) | | Untargeted MS^†^ | Reduced in indolent CLL vs. healthy:   - Sphingosine-1-phosphate - Sphinganine-phosphate   Reduced in aggressive CLL vs. healthy:   - Sphingosine-1-phosphate - Sphinganine-phosphate   No difference between indolent and aggressive CLL | Piszcz *et al.*, 2016 |
| **PBMC**  -CLL (n=5)  -Healthy (n=4) | | Targeted MS | Elevated in CLL vs. healthy:   - C24:1 GluCer - C16:0 Cer - C24:1 Cer   No statistics reported | Si Mi *et al.*, 2016 |
| **PBMC** | Targeted MS | Elevated in CLL vs. healthy: | Dielschneider *et al.*, 2016 |  |
| - CLL (n=5)  - Healthy (n=4) |  | - Sphingosine |  |  |
| **PBMC**  -CLL (n=16)  -Healthy (n=16) | | Targeted MS | Reduced CLL vs. healthy:   - 16:0 and 24:1 SM | Mayer *et al.*, 2018 |
| **PBMC**  -CLL (n=20)      M-CLL (n=6)      UM-CLL (n=6)  -Healthy (n=6) | | Targeted MS | Reduced CLL vs healthy:   - 18:1/16:0 SM and Cer - 18:1/24:1 Cer - 18:1/20:0 LacCer   Elevated CLL vs healthy:   - 18:1/22:0 SM - 18:1/16:0 GluCer - 18:1/24:1 GluCer   Reduced UM-CLL vs M-CLL   - 18:1/16:0 Cer   Elevated UM-CLL vs M-CLL   - 18:1/24:1 GluCer - 18:1/16:0 LacCer | Thurgood *et al.*, 2019 |

MS: mass spectrometry; PBMC: peripheral blood mononuclear cells; Cer: ceramide; GluCer: glucosylceramide; SM: sphingomyelins; LacCer: lactosylceramide; M-CLL : mutated IGHV; UM-CLL : unmutated IGHV.

^†^Metabolic fingerprinting using LC-MS/MS, in which 1986 metabolic features were detected.^8^

**Supplementary Table 8.** Mass spectrometry multiple reaction monitoring characteristics of sphingolipids.

| **Analyte** | **Precursor ion (*m*/*z*)** | **Product ion (*m*/*z*)** | **Retention time (min)** | **Collision energy (V)** | **Internal standard** |
| --- | --- | --- | --- | --- | --- |
| C16:0 Cer | 538.5 | 264.2 | 11.5 | 57 | C16:0 Cer-d7 |
| C24:1 Cer | 548.6 | 264.2 | 17.0 | 59 | C16:0 Cer-d7 |
| C16:0 Cer-d7 | 545.5 | 527.4 | 11.6 | 57 | - |
| C16:0 GluCer | 700.5 | 264.1 | 10.8 | 47 | d18:1 sphingosine-d7 |
| C24:1 GluCer | 810.6 | 264.1 | 14.9 | 53 | d18:1 sphingosine-d7 |
| d18:1 sphingosine | 300.3 | 282.2 | 5.06 | 17 | d18:1 sphingosine-d7 |
| d18:0 sphinganine | 302.3 | 284.2 | 5.41 | 19 | d18:1 sphingosine-d7 |
| d18:1 S1P | 380.4 | 264.2 | 6.60 | 23 | d18:1 sphingosine-d7 |
| d18:1 sphingosine-d7 | 307.4 | 289.2 | 5.10 | 17 | - |

d-Erythro-sphingosine (d18:1-sphingosine), d-Erythro-C18 Dihydro D-sphingosine (d18:0-sphinganine)

sphingosine-1-phosphate (d18:1-S1P), d-Erythro-sphingosine-d7 (d18:1-sphingosine-d7).

**Supplementary Table 10.** Primer sequences used in this study

| **qPCR** | **Forward sequence (5’ – 3’)** | **Reverse sequence (5’ – 3’)** |
| --- | --- | --- |
| *36B4 (RPLP0)* | CCCATGTGAAGTCACTGTGC | GGTTGTAGATGCTGCCATTG |
| *BAX* | CCTTTTCTACTTTGCCAGCAAAC | GAGGCCGTCCCAACCAC |
| *BIM* | ACTCTCGGACTGAGAAACGC | TTCACCTCCGTGATTGCCTT |
| *CCNA1* | TGCTCGTCACTTGGGATGGA | CGGGCTGCTGCTGGAAGA |
| *CDK1* | GCTAACATGAGAGCATGCCA | GTTCTTCCCTGTTGCGGACA |
| *EGR1* | ACCTGACCGCAGAGTCTTT | CCACAAGGTGTTGCCACTGT |
| *KI-67* | CAGGGCTGTTGATGATGGGT | CATGGTGGAAATGGGTGGGT |
| *MIR34A* | TGAGTGTTTCTTTGGCAGTGTC | CTAGGGCAGTATACTTGCTGAT |
| *PUMA* | AGAGCAGGGCAGGAAGTAAC | ACCCCATGCCAAATTTCATCC |
| *RPS6* | TGGGTGAAGAATGGAAGGGTTAT | TCCACAATGCAACCACGAAC |
| **sgRNA** | **Bottom sequence (5’ – 3’)** | **Top sequence (5’ – 3’)** |
| *UGT2B17* KO | CACCGAAGCGGAGACTGTACAGAA | AAACTTCTGTACAGTCTCCGCTTC |
| Control^†^ | CACCGACGGAGGCTAAGCGTCGCAA | AAACTTGCGACGCTTAGCCTCCGTC |
| **TIDE** | **Forward sequence (5’ – 3’)** | **Reverse sequence (5’– 3’)** |
| *UGT2B17* | GTGTTGACATCTTCGGCTTCTAT | GACTCTCTTGGTGTCCTATAGCAG |

^†^Sequences were provided by ^5^

**References for Supplementary material and methods**

1. Evans AM, DeHaven CD, Barrett T, Mitchell M, Milgram E. Integrated, nontargeted ultrahigh performance liquid chromatography/electrospray ionization tandem mass spectrometry platform for the identification and relative quantification of the small-molecule complement of biological systems. *Anal Chem*. 2009;81(16):6656-67. doi:10.1021/ac901536h

2. Dehaven CD, Evans AM, Dai H, Lawton KA. Organization of GC/MS and LC/MS metabolomics data into chemical libraries. *J Cheminform*. 2010;2(1):9. doi:10.1186/1758-2946-2-9

3. Allain EP, Rouleau M, Le T, et al. Inactivation of Prostaglandin E(2) as a Mechanism for UGT2B17-Mediated Adverse Effects in Chronic Lymphocytic Leukemia. *Front Oncol*. 2019;9:606. doi:10.3389/fonc.2019.00606

4. Heckl D, Kowalczyk MS, Yudovich D, et al. Generation of mouse models of myeloid malignancy with combinatorial genetic lesions using CRISPR-Cas9 genome editing. *Nat Biotechnol*. 2014;32(9):941-6. doi:10.1038/nbt.2951

5. Quijada-Alamo M, Hernandez-Sanchez M, Alonso-Perez V, et al. CRISPR/Cas9-generated models uncover therapeutic vulnerabilities of del(11q) CLL cells to dual BCR and PARP inhibition. *Leukemia*. 2020;34(6):1599-1612. doi:10.1038/s41375-020-0714-3

6. Brinkman EK, Chen T, Amendola M, van Steensel B. Easy quantitative assessment of genome editing by sequence trace decomposition. *Nucleic Acids Res*. 2014;42(22):e168. doi:10.1093/nar/gku936

7. Porpaczy E, Tauber S, Bilban M, et al. Lipoprotein lipase in chronic lymphocytic leukaemia - strong biomarker with lack of functional significance. *Leuk Res*. 2013;37(6):631-6. doi:10.1016/j.leukres.2013.02.008

8. Piszcz J, Lemancewicz D, Dudzik D, Ciborowski M. Differences and similarities between LC-MS derived serum fingerprints of patients with B-cell malignancies. *Electrophoresis*. 2013;34(19):2857-64.
